# On-going Mechanical Damage from Mastication Drives Homeostatic Th17 Cell Responses at the Oral Barrier

**DOI:** 10.1016/j.immuni.2016.12.010

**Published:** 2017-01-17

**Authors:** Nicolas Dutzan, Loreto Abusleme, Hayley Bridgeman, Teresa Greenwell-Wild, Tamsin Zangerle-Murray, Mark E. Fife, Nicolas Bouladoux, Holly Linley, Laurie Brenchley, Kelly Wemyss, Gloria Calderon, Bo-Young Hong, Timothy J. Break, Dawn M.E. Bowdish, Michail S. Lionakis, Simon A. Jones, Giorgio Trinchieri, Patricia I. Diaz, Yasmine Belkaid, Joanne E. Konkel, Niki M. Moutsopoulos

**Affiliations:** 1 Oral Immunity and Inflammation Unit, NIDCR, NIH, Bethesda, MD 20892, USA; 2 Faculty of Biology, Medicine and Health, University of Manchester, Manchester M13 9PT, UK; 3 Manchester Collaborative Centre for Inflammation Research (MCCIR), University of Manchester, Manchester M13 9NT, UK; 4 Immunity at Barrier Sites Initiative, NIAID, NIH, Bethesda, MD 20892, USA; 5 Mucosal Immunology Section, Laboratory of Parasitic Diseases, NIAID, NIH, Bethesda, MD 20892, USA; 6 Fungal Pathogenesis Unit, NIAID, NIH, Bethesda, MD 20892, USA; 7 Department of Pathology and Molecular Medicine, McMaster University, Hamilton, ON L8N 3Z5, Canada; 8 Institute of Infection and Immunity, School of Medicine, Cardiff University, Cardiff CF14 4XN, UK; 9 Cancer and Inflammation Program, Center for Cancer Research, NCI, NIH, Bethesda, MD 20892, USA; 10 Division of Periodontology, Department of Oral Health and Diagnostic Sciences, UConn Health Center, Farmington, CT 06030, USA

**Keywords:** mucosal immunology, Th17 cells, IL-17, oral immunity, barrier immunity, periodontitis, T cells

## Abstract

Immuno-surveillance networks operating at barrier sites are tuned by local tissue cues to ensure effective immunity. Site-specific commensal bacteria provide key signals ensuring host defense in the skin and gut. However, how the oral microbiome and tissue-specific signals balance immunity and regulation at the gingiva, a key oral barrier, remains minimally explored. In contrast to the skin and gut, we demonstrate that gingiva-resident T helper 17 (Th17) cells developed via a commensal colonization-independent mechanism. Accumulation of Th17 cells at the gingiva was driven in response to the physiological barrier damage that occurs during mastication. Physiological mechanical damage, via induction of interleukin 6 (IL-6) from epithelial cells, tailored effector T cell function, promoting increases in gingival Th17 cell numbers. These data highlight that diverse tissue-specific mechanisms govern education of Th17 cell responses and demonstrate that mechanical damage helps define the immune tone of this important oral barrier.

## Highlights

• Distinct signals shape the Th17 cell network at the oral barrier
• Oral barrier Th17 cells develop independently of commensal microbe colonization
• Physiologic damage through mastication promotes the generation of oral Th17 cells
• Barrier damage triggers oral Th17-cell-mediated protective immunity and inflammation

## Graphical Abstract

**Figure:**
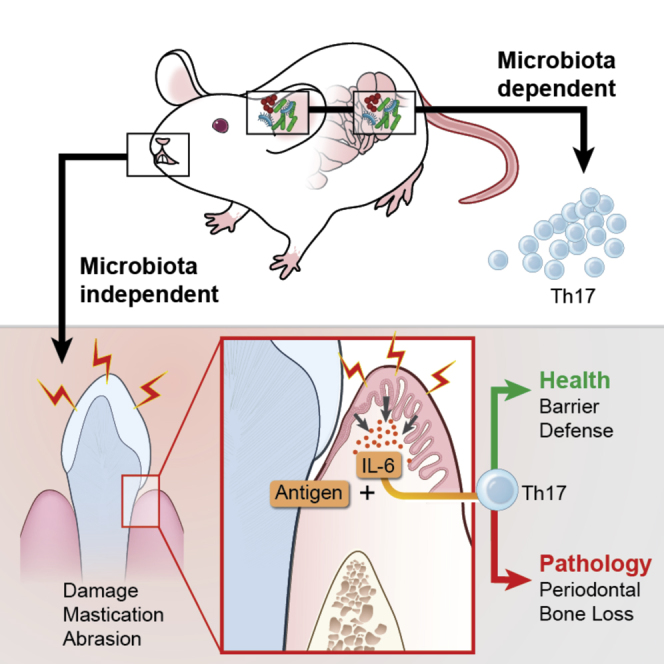

## Teaser

The signals regulating immunity at the gingiva, a key oral barrier, remain unclear. Dutzan et al. show that oral barrier Th17 cells are induced in response to mastication rather than commensal colonization, identifying physiologic mechanical damage as a unique tissue-specific cue conditioning local immunity and inflammation at the oral barrier.

## Introduction

Barrier-resident immune populations integrate local cues to generate responses that preserve barrier integrity, maintain host-commensal interactions, and aid in fighting infection (Cash et al., 2006, Franchi et al., 2012). In recent years our understanding of barrier-tailoring of immune responses has dramatically expanded. This is particularly true in the gastrointestinal (GI) tract and skin, where tissue-specific and microbial-derived signals have been shown to shape the immune surveillance network and immune responsiveness (Ivanov et al., 2009, Naik et al., 2012, Smith et al., 2013). Yet, little is known regarding the development of tissue-specific immunity at the gingiva, an essential oral barrier that supports the dentition, harbors a complex commensal microbiome, and is a site where food antigens are first encountered prior to GI tract entry. Indeed, how effective immunity and regulation are balanced at this oral barrier is poorly understood. Expanding our understanding of the basic mechanisms controlling immunity at this barrier is important because the breakdown of controlled immune responses at the gingiva leads to periodontitis, a common inflammatory disease of humans. Additionally, periodontitis has been linked to the potentiation of a plethora of inflammatory conditions, such as cardiovascular disease and rheumatoid arthritis (Hajishengallis, 2015). Therefore, understanding the mediators of health and disease at the gingiva may have broad-reaching implications for systemic inflammation.

T helper 17 (Th17) cells are key mediators of barrier immunity, participating in immune surveillance and maintenance of barrier integrity (Weaver et al., 2013). Importantly, this T cell subset has been implicated in mediating protective immunity as well as pathogenic inflammation at the oral barrier. The development of Th17-cell-mediated responses at barriers such as the skin and GI tract is linked to tissue-specific factors, particularly colonization by site-specific commensals (Ivanov et al., 2009, Naik et al., 2012). However, in the gingiva the factors controlling tissue-specific immunity remain ill defined, and as such it is not known how Th17 cells are induced in this environment. The critical role of Th17 cells in mediating protection at the oral barrier is evident in patients with genetic defects in Th17 cell differentiation and function; these patients present with severe and recurrent oral fungal infections (Liu et al., 2011, Moutsopoulos et al., 2015). However, exaggerated Th17 cell responses at the gingiva are detrimental and have been shown to promote inflammatory bone loss and tissue damage in periodontitis (Eskan et al., 2012, Moutsopoulos et al., 2014). How Th17 cells are induced in the gingiva and subsequently become deregulated in periodontitis is poorly understood. Therefore, elucidating the factors involved in the induction and regulation of Th17 cells in this environment will shed light on the tissue-specific cues that regulate immunity in the gingiva.

Here we delineated the mechanisms controlling accumulation of Th17 cells in the gingiva. Our data show that the gingival interleukin 17 (IL-17)-producing CD4^+^ T cell population increased with age. Exploring this increase in Th17 cells in older mice, we found that the mechanisms controlling CD4^+^ T cell effector function in the gingiva were different to those operating at other barrier sites. Our data demonstrate that gingival Th17 cells were not dependent on colonization by commensal bacteria, as the Th17 cell population was unchanged in germ-free mice. However, gingival Th17 cells were dependent upon IL-6-mediated signals. We identified that mechanical damage, which induces IL-6 and occurs physiologically in the oral cavity through mastication and abrasion, promoted accumulation of gingival Th17 cells. Thus, damage, as opposed to commensal colonization, helps define the immune tone of the gingiva, clearly demonstrating that unique rules shape gingival immune-homeostasis.

## Results

### Gingiva Th17 Cell Frequencies Increase with Age

**Figure 1:**
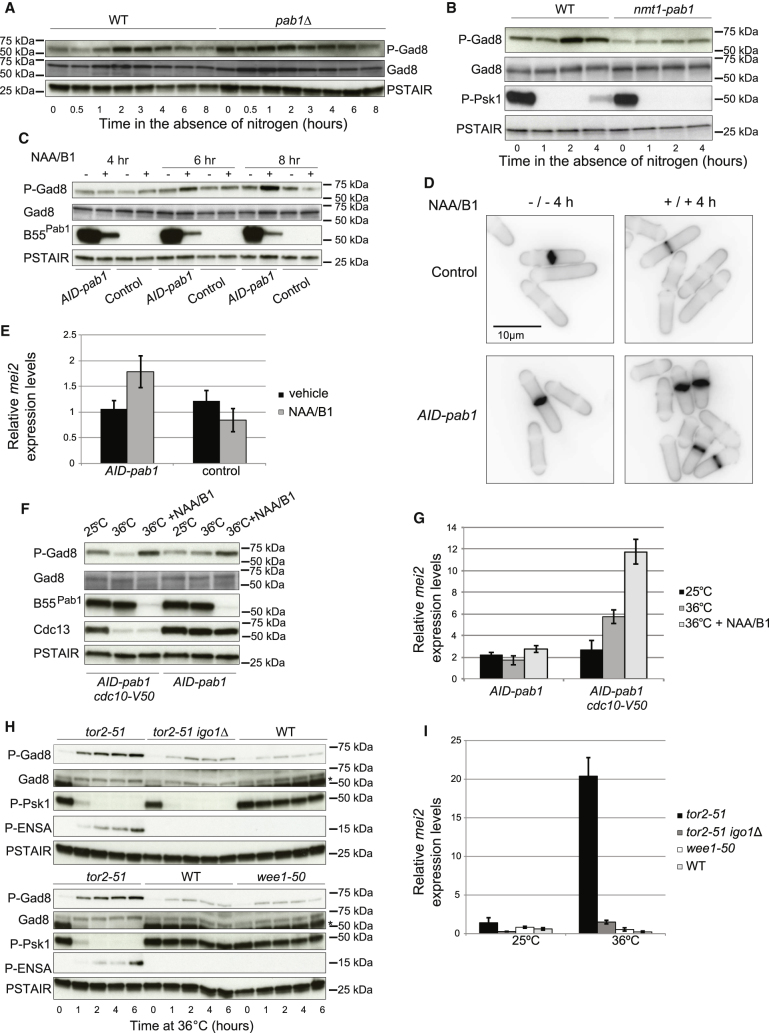
Frequencies of Oral Barrier IL-17-Producing CD4^+^ T Cells Increase with Age (A–D) Single-cell preparations of mouse gingiva were stimulated with PMA and ionomycin. ^∗^p < 0.05, ^∗∗^p < 0.005 as determined by one-way ANOVA. (A and B) Representative FACS plots show IFN-γ versus IL-17 staining gated on CD4^+^ T cells in the gingiva of (A) 8- and (B) 24-week-old mice. Numbers in gates indicate percentages of cells. (C) Bar graphs show frequencies of gingiva CD4^+^ T cells producing IFN-γ (left) and IL-17 (right). (D) Bar graph shows number of gingiva IL-17^+^CD4^+^ T cells. n = 6–28; data from 4+ experiments. (E) Bar graphs show frequencies of CD4^+^ T cells producing IL-17 in the small intestinal lamina propria (SI Lp), oral barrier draining lymph node (LN), and spleen of 8-week-old (n = 4–5; white bars) and 24-week-old (n = 5–12; black bars) mice. (F) Bar graph shows frequency of CD4^+^Foxp3^+^ T cells in the gingiva of 8-week-old (n = 6) and 24-week-old (n = 4) mice, examined over three experiments. (G) Bar graphs show the percent of gingival IL-17^+^ or IFN-γ^+^ cells that are positive for Ki67 (left) or Bcl-2 (right) from 8-week-old (n = 7–9; white bars) and 24-week-old (n = 10; black bars) mice. Data from three separate experiments. (H–J) Single-cell preparations of human gingiva were stimulated with PMA and ionomycin. (H) Representative FACS plots show IFN-γ versus IL-17 staining on live, CD45^+^ cells in gingiva of healthy individuals who were 18–25 years of age or 40–50 years of age. (I) Representative FACS plots further characterizing the IL-17^+^ population in human gingiva; there was little staining for TCRγδ within the IL-17^+^ population (Figures S1D and S1E). Numbers in gates indicate percentages of cells. (J) Graph showing frequency of gingival IL-17^+^ cells in healthy individuals who were 18–25 (n = 9) or 40–50 years of age (n = 10). ^∗^p < 0.05 as determined by unpaired Student’s t test. Error bars represent mean ± SEM. See also Figure S1.

In order to understand local induction of Th17 cell responses, we examined IL-17^+^ T cells in mouse gingiva, the mucosal tissue surrounding the dentition and key oral barrier in periodontitis. At steady-state Th17 cells are enriched at barrier sites, specifically the GI tract and skin (Ivanov et al., 2008, Naik et al., 2012). In contrast, few Th17 cells were seen in the gingiva of 8-week-old (young) mice (Figure 1A). However by 24 weeks of age, considered middle age in aging studies, Th17 cell frequencies (Figures 1B and 1C) and numbers (Figure 1D) were significantly elevated in the gingiva, indicating the physiologic development of a Th17 cell network with age.

This increase in Th17 cells at 24 weeks was specific to the gingiva and was not seen at other barriers, the oral-draining lymph node, or spleen (Figure 1E). This age-dependent expansion was also unique to Th17 cells, as gingiva from 24-week-old mice exhibited reduced frequencies, although not total numbers, of interferon γ (IFN-γ)-producing T cells (Figures 1B and 1C) and an unchanged regulatory T (Treg) cell network (Figure 1F). Therefore, unlike other barrier sites, the gingiva showed a remodelled cytokine network during aging. This natural, age-driven increase in Th17 cells provided the ideal setting for us to probe the development of disease-relevant gingival Th17 cells.

We first determined that these cells required antigen for their development, as they were absent in 24-week-old T cell receptor (TCR)-transgenic animals (Figure S1A). Next we assessed whether enhanced proliferation or survival of Th17 cells could be contributing to the enlarged gingival Th17 cell population with age. Staining of gingiva Th17 cells from 8- and 24-week old mice for the proliferation marker Ki67 and the anti-apoptotic marker B cell Lymphoma 2 (Bcl-2) showed that increased proliferation, not survival, contributed to the enlarged gingival Th17 cell population that emerged with age (Figure 1G).

We extended these observations to human gingiva by evaluating IL-17^+^ cell frequencies in younger (18–25 years of age) and older (40–50 years of age) healthy volunteers with no evidence of periodontitis (Eke et al., 2012) or other oral disease (Figure S1B). We saw increased frequencies of IL-17^+^ cells in the gingiva of older compared to younger adults (Figures 1H–1J). No increases in IFN-γ^+^ cells were seen (Figure S1C), and thus our data demonstrated an age-dependent, gingival-specific expansion of Th17 cells.

### Shifts in Microbial Communities Do Not Correlate with Th17 Cell Development

**Figure 2:**
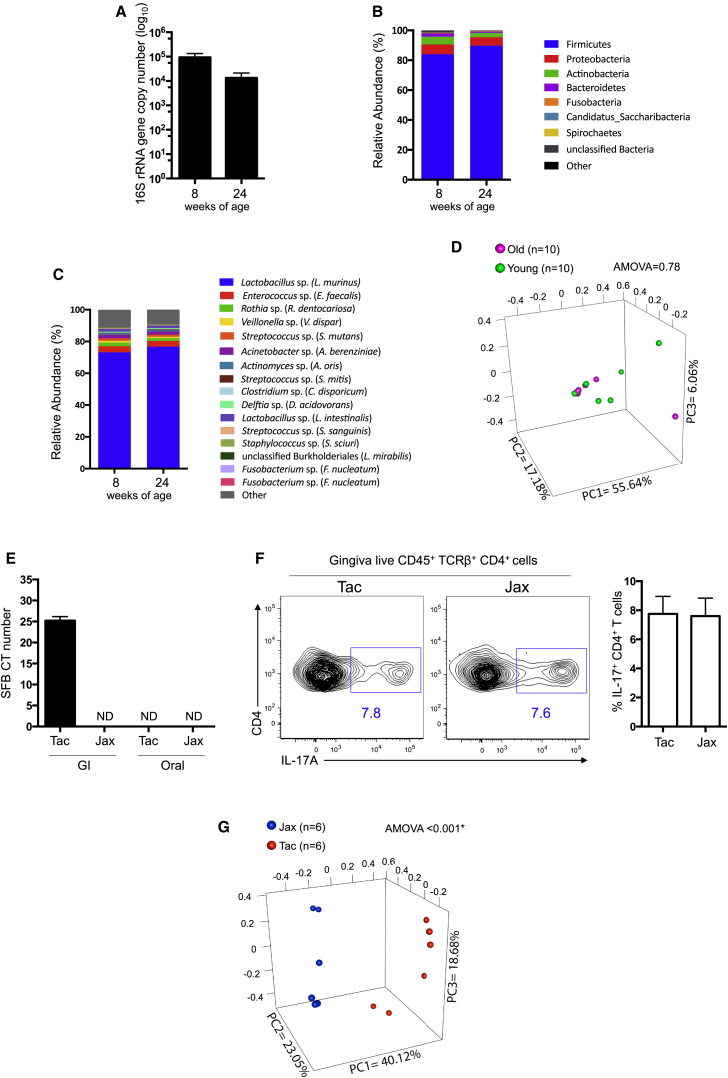
Microbiome Shifts Do Not Correlate with the Presence of Gingival Th17 Cells (A) Graph shows comparison of total bacterial load in the oral cavity of 8- and 24-week-old mice, determined by a 16S rRNA-based real-time PCR assay. (B and C) Graphs show microbiome composition at different taxonomical levels, depicting most abundant (B) phyla and (C) OTUs in longitudinally sampled mice (n = 10). No differences in relative abundances were observed between young and old mice. (D) PCoA plot based on thetaYC distances showing no difference in global community structure at the 8- and 24-week time points (n = 10). Some data points are not visible due to tight clustering. (E) SFB levels in cecum samples and oral swabs of mice from Taconic Farms (Tac) and Jackson Laboratories (Jax). Bar graph shows CT value for the real-time PCR reaction, ND indicates below the level of detection for the assay. (F) Representative FACS plots show CD4 verses IL-17 staining gated on gingiva CD45^+^TCRβ^+^CD4^+^ T cells from either 24-week-old Tac (n = 12) or Jax (n = 4) mice. Bar graph shows frequency of gingiva IL-17^+^CD4^+^ T cell in Tac and Jax mice from two separate experiments. (G) PCoA plot based on thetaYC distances showing Tac and Jax mice cluster apart, indicating different oral microbiomes. p < 0.001 as determined by AMOVA. Error bars represent mean ± SEM. See also Figure S2.

Commensal communities shape tissue immunity in health and disease and specific oral microbes are implicated in the development of not only periodontitis (Abusleme et al., 2013, Griffen et al., 2012) but also distinct peripheral pathologies (Koren et al., 2011, Kostic et al., 2012). Commensal bacteria play vital roles in Th17 cell development at other barriers (Naik et al., 2012), with specific species driving Th17 cell development (Ivanov et al., 2009). Therefore, we first investigated whether changes in oral microbial communities could account for elevated gingival Th17 cells with age. We found no significant differences in bacterial biomass, diversity, or composition in mice at 8 versus 24 weeks of age (Figures 2A–2D). These data allowed us to undertake a detailed examination of the mouse oral microbiome, revealing Firmicutes as the dominant phylum (Figure 2B) and *Lactobacillus* having the most abundant operational taxonomic units (OTUs) in the oral cavity (Figure 2C). Importantly, some OTUs detected in lower abundance were closely related to “signature” species of the human oral microbiome (Aas et al., 2005, Abusleme et al., 2013), including *Veillonella dispar*, *Rothia dentocariosa*, *Streptococcus mutans*, and *Actinomyces oris*, suggesting conserved oral microbiome elements in humans and mice.

Next, we specifically interrogated the presence of segmented filamentous bacteria (SFB), which promote generation of Th17 cells in the GI tract (Ivanov et al., 2009). These key Th17-cell-driving bacteria were not constituents of the oral microbiome (Figure 2E). However, GI colonization by SFB can support Th17 cell generation at peripheral sites (Lee et al., 2011, Wu et al., 2010). Therefore we examined Th17 cells in the gingiva of 24-week-old SFB^+^ mice from Taconic (Tac) and SFB^−^ mice from Jackson Laboratories (Jax). No difference in the frequencies of gingival Th17 cells in SFB^+^ and SFB^−^ mice were seen (Figure 2F), demonstrating that gingival Th17 cell development was independent of SFB. Moreover, despite similar Th17 cell frequencies, Tac and Jax mice had significant differences in their oral bacterial communities (Figures 2G and S2), further suggesting that the oral microbiome may not be a primary driver of gingival Th17 cell development.

### Gingival Th17 Cells Arise Independently of Commensal Colonization

**Figure 3:**
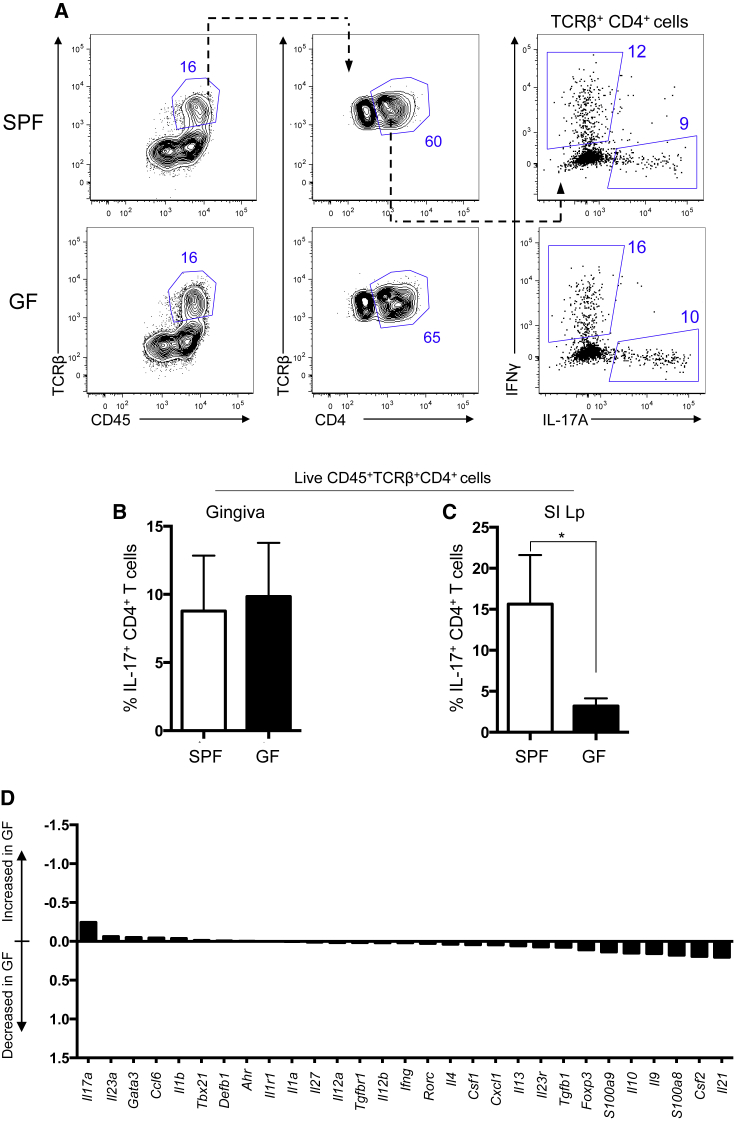
Th17 Cell Accumulation at the Oral Barrier Occurs Independently of Commensal Colonization (A and B) Th17 cell frequencies were examined in age-matched SPF and GF mice. (A) Representative FACS plots show gating for CD4^+^ T cells in gingiva. Right plots show IFN-γ versus IL-17 staining in live, CD4^+^ T cells. Top row, SPF mice; bottom row, GF mice. Numbers in gates indicate percentages of cells. (B) Bar graph shows frequency of gingiva IL-17^+^CD4^+^ T cell in aged-matched SPF (n = 6) and GF (n = 7) mice from 3 experiments. (C) Bar graph shows frequency of small intestine lamina propria (SI Lp) IL-17^+^CD4^+^ T cell in SPF (n = 5) and GF (n = 5) mice from 2 experiments. (D) Bar graph shows log fold change in expression of indicated genes in GF relative to SPF gingiva. Data representative of two independent nano-string runs with a total of four samples per group. Error bars represent mean ± SEM. See also Figure S3.

To fully evaluate the role of commensal bacteria in promoting gingival Th17 cells, we examined these cells in age-matched germ-free (GF) and specific-pathogen-free (SPF) mice. In the skin and GI tract, barrier-resident Th17 cells are dramatically reduced in GF mice (Ivanov et al., 2009, Naik et al., 2012), demonstrating that at these barriers Th17 cells are dependent upon commensal bacteria colonization. However, this was not the case in the gingiva, where similar frequencies and total numbers of Th17 cells were seen in both GF and SPF mice (Figures 3A, 3B, and S3A). This differed from what was seen in the GI tract (Figure 3C) and shows that in contrast to other barrier sites, Th17 cell accumulation in the gingiva occurred independently of bacterial colonization.

Accumulation of Th17 cells at the gingiva also did not occur in response to fungal recognition, as gingival Th17 cells were unchanged in the absence of Dectin-1 and Mannose receptor signaling (Figures S3B and S3C). Broader evaluation of the immune signatures of GF and SPF gingiva revealed that expression of genes known to affect Th17 cell generation (e.g., *tgfb1* and *il1b*), downstream of IL-17 (e.g., *s100a9*, *s100a8*, *csf2*), and part of the IL-17 signature (e.g., *rorc*, *il17a*) were similarly expressed in SPF and GF gingiva (Figure 3D). Frequencies of CD45^+^ cells and T cells in the gingiva were also unchanged in GF mice compared to control mice (Figure S3D). Moreover, the frequencies of gingival Treg cells were similar in GF and SPF mice (Figure S3E).

In sum our data show that, in contrast to other barrier sites, bacterial colonization was not required to promote the physiological accumulation of Th17 cells in the gingiva, highlighting that unique factors ensure that Th17 cells populate this barrier.

### Gingival Th17 Cells Are Dependent upon IL-6

**Figure 4:**
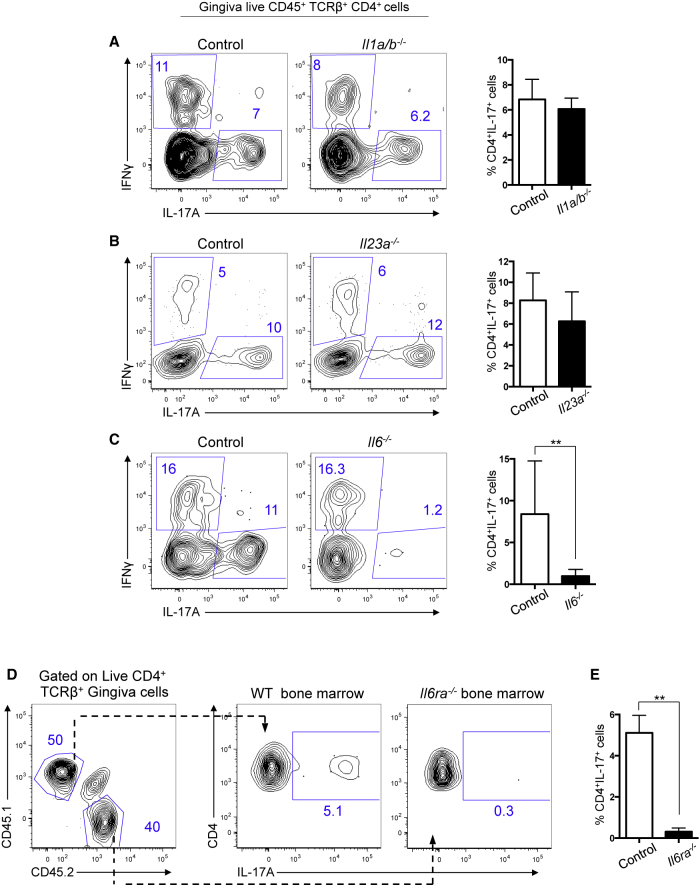
Differentiation of Oral Barrier Th17 Cells Is Dependent upon IL-6 (A–C) Representative FACS plots showing IFN-γ versus IL-17 staining gated on gingiva CD45^+^TCRβ^+^CD4^+^ T cells from age-matched old control or gene-deficient animals and bar graphs show frequency of gingival CD4^+^IL-17^+^ cells in (A) control (n = 8) and *il1a* and *il1b*^−/−^ double gene-deficient (*il1a/b*^*−/−*^) (n = 7) mice, (B) control (n = 4) and *il23a*^−/−^ (n = 4) mice, and (C) control (n = 7) and *il6*^−/−^ (n = 9) mice, examined over 2–4 experiments. (D and E) Chimeric mice comprised of wild-type CD45.1^+^ and *il6ra*^−/−^ CD45.2^+^ bone marrow were generated in CD45.1^+^CD45.2^+^ hosts and gingiva CD4^+^ T cell cytokine production examined at 24 weeks of age. (D) Representative FACS plot show CD45.1 and CD45.2 staining on gated CD4^+^ T cells and IL-17 staining in wild-type and *il6ra*^−/−^ T cells in the same mouse. Numbers in gates indicate percentages of cells. (E) Bar graph shows frequency of gingival IL-17^+^CD4^+^ T cells in control and *il6ra*^−/−^ bone marrow compartments. Data representative of two independent experiments with six to eight mice/group. ^∗∗^p < 0.005 as determined by unpaired Student’s t test. Error bars represent mean ± SEM. See also Figure S4.

Next we sought to determine the cytokine cues required for accumulation of gingival Th17 cells. We first examined a role for IL-1 and IL-23, cytokines that promote the Th17 cell phenotype in naive CD4^+^ T cells (Harrington et al., 2005, McGeachy et al., 2009) and are key for the development and maintenance of Th17 cells in the GI tract and skin (Coccia et al., 2012, Naik et al., 2012, Shaw et al., 2012). IL-1 and IL-23 were dispensable for gingival Th17 cells (Figures 4A, 4B, S4A, and S4B) as shown by the fact cytokine-deficient animals, specifically *il1a/b*^−/−^ (*il1a* and *il1b* double-deficient mice; Figure 4A) and *il1r1*^−/−^ (Figure S4A) as well as *il23a*^−/−^ (Figure 4B) and *il12b*^−/−^ (Figure S4B) mice exhibited unchanged frequencies of gingival Th17 cells.

IL-6 also promotes Th17 cell differentiation (Bettelli et al., 2006, Mangan et al., 2006, Veldhoen et al., 2006). We found that development of gingival Th17 cells was dependent on IL-6 as Th17 cells were drastically reduced in the gingiva of *il6-*deficient animals (Figure 4C). To understand whether the requirement for IL-6 signals was intrinsic or extrinsic to T cells, we generated mixed bone marrow chimeras by combining congenically marked wild-type and *Il6ra*^*−/−*^ (lacking expression of the IL-6R) bone marrow. Examining gingiva CD4^+^IL-17^+^ T cells in these chimeras demonstrated that gingival T cells had a cell-intrinsic requirement for IL-6 signaling to produce IL-17, as *Il6ra*^*−/−*^ CD4^+^ T cells in the gingiva did not make IL-17 but wild-type CD4^+^ T cells in the same environment did (Figures 4D and 4E). In contrast, both wild-type and *Il6ra*^*−/−*^ CD4^+^ T cells in the skin and GI tract of these chimeras could make IL-17 (Figure S4C).

These data indicate that distinct signals support Th17 cells in the gingiva compared to those in operation at other barrier sites, with Th17 cells accumulating in the gingiva independently of commensal colonization and in an IL-6-dependent manner.

### Physiological Mechanical Damage Promotes Gingival Th17 Cells

**Figure 5:**
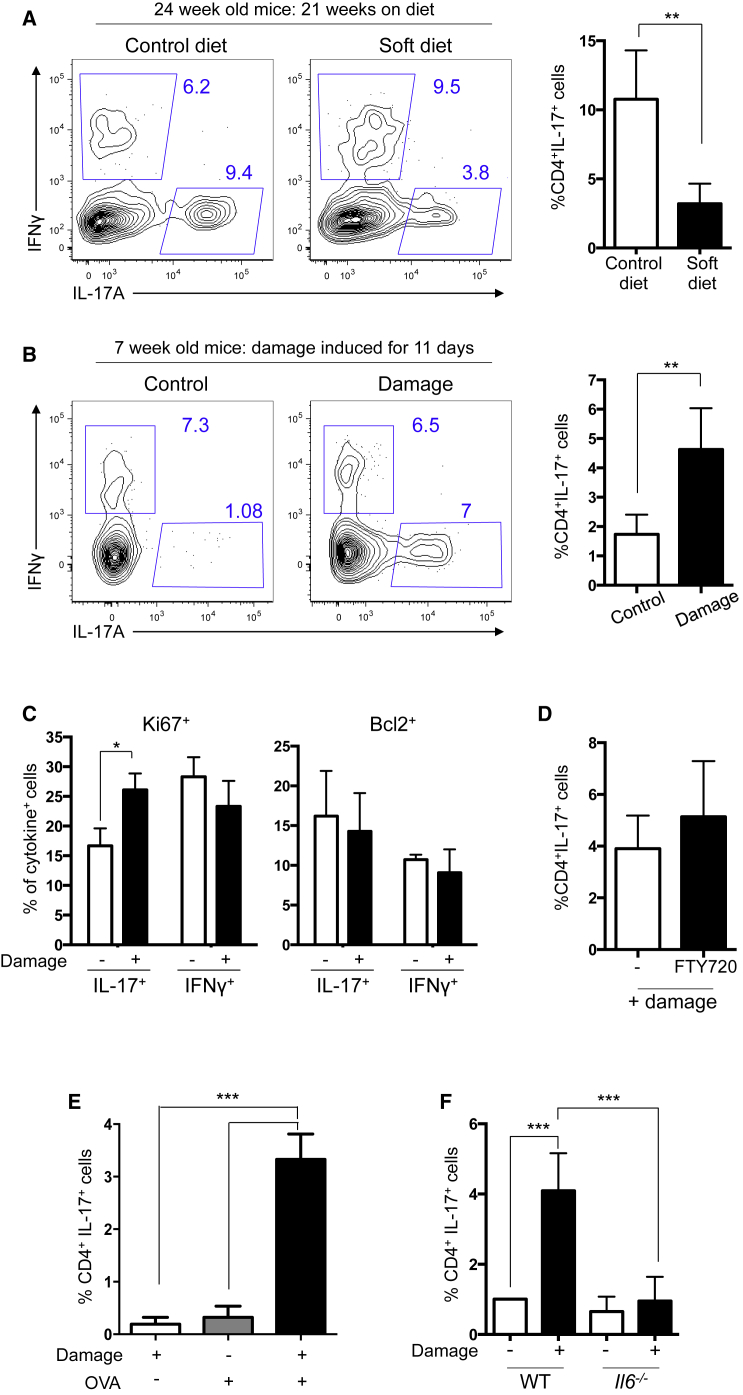
Oral Barrier Damage Drives Generation of Gingival Th17 Cells (A) FACS plots show IFN-γ versus IL-17 staining in gingival CD45^+^TCRβ^+^CD4^+^ T cells from 24-week-old mice fed control or soft diet from weaning. Data are from three experiments with two to three mice/group. (B) FACS plots show IFN-γ versus IL-17 staining in gingival CD45^+^TCRβ^+^CD4^+^ T cells from young control or age-matched mice that experienced gingival damage every other day for 11 days. (C) Bar graphs show frequency of gingival IL-17^+^ or IFN-γ^+^ cells positive for Ki67 (left) or Bcl-2 (right) from control mice (−; white bars) or mice that experienced repeated gingival damage (+; black bars). Data are from two to three separate experiments with three to four mice/group. (D) Young mice underwent gingival barrier damage every other day for 11 days and at the same time received either FTY720 (black bars) or saline (white bars) i.p. Bar graph shows frequency of gingival CD4^+^IL-17^+^ cells. Data from two separate experiments with two to three mice/group. (E) OT-IIxRag^−/−^ mice were either (1) not exposed to OVA but experienced gingival damage, (2) exposed to OVA ad libitum in the drinking water (1.5%) and topically at the gingiva (1 mg/mouse every other day), or (3) exposed to OVA ad libitum in the drinking water (1.5%) and topically at the gingiva (1 mg/mouse every other day) and also experienced gingival barrier damage. Gingival tissues were examined for Th17 cells at day 10. Bar graph shows percent of gingival IL-17^+^CD4^+^ T cells. Data are representative of two experiments with three to four mice/group. (F) Young, age-matched control or *il6*^*−/−*^ mice were left untreated (−; white bars) or experienced gingival barrier damage every other day for 11 days (+; black bars) after which Th17 cells were examined. Bar graph shows percent of gingiva IL-17^+^CD4^+^ T cells. Data representative of two experiments with two to four mice/group. ^∗^p < 0.05, ^∗∗^p < 0.01 as determined by unpaired Student’s t test. ^∗∗∗^p < 0.05 as determined by one-way ANOVA. Error bars represent mean ± SEM. See also Figure S5.

We next addressed how gingival Th17 cells could develop independently of endogenous commensal bacteria. A unique tissue-specific signal present in the oral environment is on-going mastication. Mastication requires mechanical force and leads to local barrier abrasion and damage. We queried whether mastication was a physiologic stimulus contributing to the tailoring of gingival T cell function. We addressed this by altering levels of these stimuli and then examining gingival Th17 cells. First, we reduced the mechanical forces of mastication on the oral barrier by placing weanling mice on nutritionally matched soft diets. Mice remained on this diet until 24 weeks of age when gingiva Th17 cells were assessed. Reduction in the physiological stimuli induced by mastication resulted in a significant decrease in gingiva Th17 cells (Figures 5A and S5A). This occurred specifically in the gingiva and not in the local draining lymph node (Figure S5B), suggesting that mastication locally supports gingival Th17 cells.

To directly assess whether local barrier damage was a stimulus promoting gingival Th17 cells, we increased the levels of damage at the gingiva of young mice, in which few Th17 cells were seen (Figure 1). Gingival damage was enhanced by increasing levels of barrier abrasion, through rubbing of the gingiva with a sterile cotton applicator once every other day for 11 days. This direct induction of mechanical damage resulted in increased frequencies (Figure 5B) and numbers (Figure S5C) of gingival Th17 cells. Th17 cell increases were not seen in draining lymph nodes, further underscoring the compartmentalized nature of this response (Figure S5D).

We next wanted to understand the mechanism(s) by which gingival damage promoted increases in the number of gingival barrier Th17 cells. We assessed whether the increased gingival Th17 cells were due to elevated IL-17^+^ T cell recruitment, proliferation, or survival. In line with our data from aged mice, where damage occurs physiologically over time due to mastication (Figure 1G), in our damage-induction model, gingival IL-17^+^CD4^+^ T cells showed greater proliferation but no change in pro-survival factor expression (Figure 5C). To examine whether elevated recruitment of Th17 cells after damage could also play a role, we transferred in vitro differentiated Th17 and Th0 cells and examined Th17 cell recruitment to the gingiva. Th17 cells were not recruited to the gingiva to a greater degree than Th0 cells either before or after damage (Figures S5E and S5F). These data, along with data demonstrating that the lymph node egress inhibitor FTY720 did not alter the gingival Th17 cell population after damage (Figure 5D), suggested that elevated recruitment did not contribute to the increased gingival Th17 cell frequencies arising after damage. Combined, our data indicate that damage promotes the proliferation of gingival IL-17^+^ T cells.

Next we determined whether damage-induced expansion of gingival Th17 cells required antigen recognition. We found that increased frequencies of gingival Th17 cells were not seen in response to damage in the absence of cognate antigen, demonstrating a requirement for both damage and antigen in promoting an enlarged population of gingival Th17 cells (Figure 5E). In response to gingival damage, *il6*^−/−^ animals failed to show an increased population of gingival Th17 cells (Figure 5F), outlining a vital role for IL-6 in this damage-induced process. Combined, our data demonstrate that local physiological mechanical damage to the gingiva modulates the gingival barrier T cell network, promoting Th17 cells in an IL-6- and antigen-dependent manner.

Although mechanical damage occurs physiologically at the gingiva, we queried whether repeated damage to other barriers could promote increases in local Th17 cells. We show this was the case after repeated skin damage (Figure S5G), revealing the activity of this pathway even at a site where Th17 cells are dominantly educated by commensals.

### Gingiva Damage Rapidly Induces IL-6 from Epithelial Cells in a Commensal-Independent Manner

**Figure 6:**
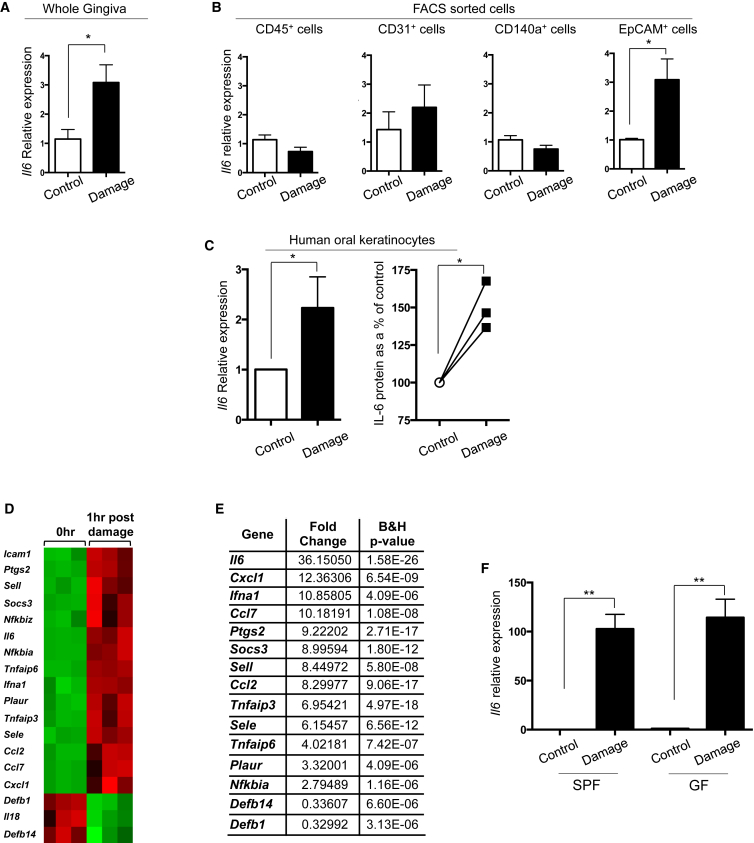
Gingival Damage Induces Rapid Production of IL-6 from Epithelial Cells (A) At day 11 after gingival damage, *il6* expression was determined by qPCR in dissected gingival tissues of young mice. *il6* expression is shown for samples from mice experiencing damage relative to that in control gingiva. Graph represents data from five mice/group. (B) CD45^+^ and different populations of CD45^−^ cells were FACS sorted from the gingiva of control mice (white bars) or mice that experienced gingival damage 4 hr prior (black bars). Sorted populations were CD45^+^ cells, endothelial cells (CD45^−^CD31^+^), fibroblasts (CD45^−^CD31^−^EpCAM^−^CD140a^+^), and epithelial cells (CD45^−^CD31^−^EpCAM^+^). Bar graphs show *il6* expression determined by qPCR and is shown in cells sorted from damaged gingiva relative to that in controls. Data are from two to three separate FACS sorts. (C) In vitro scratch assays on human oral keratinocytes cells. Left: RNA expression examined after 4 hr; graph shows *il6* levels in damaged cells relative to that in un-damaged control. Right: Graph shows IL-6 levels in HOK supernatants examined 18–24 hr after scratching and presented as percent of control. Data from three experiments. (D and E) Nanostring immune gene array was performed on gingival tissues from mice that had experienced damage or controls. (D) Heatmap of differentially regulated genes (adjusted; p < 0.001). (E) List of 15 most upregulated genes in murine gingiva 1 hr after damage. Data from three replicates. (F) Young, age-matched SPF and GF mice were left untreated (control) or experienced gingival barrier damage (damage). 1 hr later gingival tissues were harvested and *il6* expression examined by qPCR. Expression is shown in damaged gingiva relative to untreated controls (n = 5 mice/group). ^∗^p < 0.05, ^∗∗^p < 0.0003, as determined by unpaired Student’s t test. Error bars represent mean ± SEM. See also Figure S6.

Consistent with the IL-6 dependency of gingiva Th17 cells, IL-6 was elevated in the gingiva after mechanical damage (Figure 6A). Next we identified the cellular source of IL-6 after damage by initially FACS sorting gingival CD45^−^ and CD45^+^ cells. Only CD45^−^ cells showed increased *il6* mRNA levels after damage (Figure S6A). To define the source of IL-6, we FACS purified endothelial cells, fibroblasts, and epithelial cells, as well as remaining CD45^−^ cells and CD45^+^ cells, after gingival damage (Figure S6B). In response to damage, *il6* transcription was elevated only in epithelial cells (Figure 6B). This damage-induced IL-6 from epithelial cells appeared to be a conserved response (Zhang et al., 2015), as we saw increased *il6* messenger RNA (mRNA) and protein after damage of human oral epithelial cells (HOK cells) in vitro (Figure 6C).

Consistent with an IL-6-dependent development of gingival Th17 cells, levels of *il6* mRNA in bulk gingival CD45^−^ cells correlated with Th17 cell frequencies, with higher expression levels in 24- versus 8-week-old mice, and similar expression levels in gingival CD45^−^ cells from age-matched GF and SPF mice (Figures S6C and S6D).

Increased *il6* expression occurred rapidly within 1 hr of barrier damage (Figures 6D and S6E). Moreover, transcriptomic analysis of immune genes upregulated within 1 hr of gingival damage revealed that *il6* was the most highly upregulated gene (Figures 6D and 6E). Pathway analysis of array data from in vivo damaged gingival tissue and in vitro damaged human oral keratinocytes showed activation of the IL-6-signaling pathway, as well as the NF-κB-signaling pathway, which is implicated in *il6* transcription (Figure S6F; Libermann and Baltimore, 1990). Inhibition of NF-κB signaling in vitro led to a reduced upregulation of *il6* mRNA after damage, suggesting some role for NF-κB in damage-induced *il6* activation (Figure S6G).

Finally, we queried whether rapid *il6* upregulation after gingival damage was influenced by commensal bacteria. Increases in *il6* mRNA after damage were seen in both SPF and GF animals and were increased to the same extent in both sets of mice (Figure 6F). Combined, these data demonstrate that local mechanical damage to the gingiva induces rapid production of IL-6 from epithelial cells, which is subsequently vital for gingival Th17 cells.

### Damage-Induced Responses Contribute to Protective Immunity and Inflammation at the Gingiva

**Figure 7:**
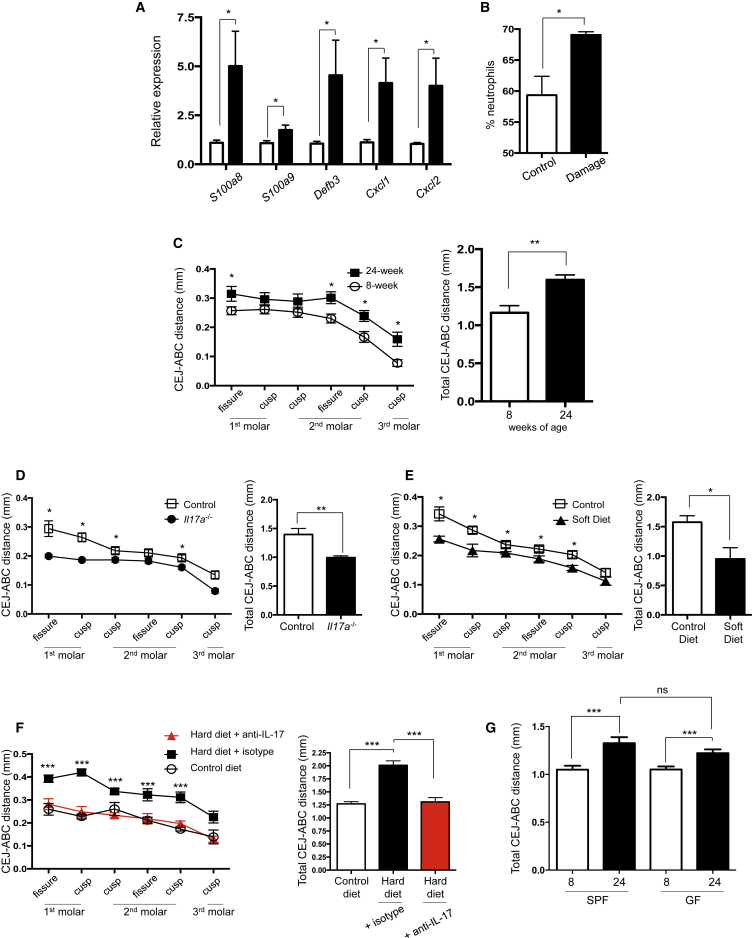
Gingival Damage Amplifies Oral Immune Responsiveness (A and B) Gingival tissues were isolated from mice that experienced gingival damage every other day for 11 days followed by 5–10 days of rest. (A) Graph shows gene expression in gingival tissues of mice experiencing damage relative to controls. Data are from three experiments with two to five mice/group. (B) Bar graph shows frequencies of neutrophils in gingival CD45^+^MHCII^−^ cells and shows data from two experiments. (C) Cemento-enamel junction (CEJ) to alveolar bone crest (ABC) distances in maxilla of 24-week-old (closed squares; n = 5) and 8-week-old (open circles; n = 5) mice. Left: CEJ-ABC distance was measured at six defined points across the molars. Right: Graph shows total CEJ-ABC distance. (D) CEJ to ABC distances in maxilla of 24-week-old wild-type (open squares; n = 9) or *il17a*^−/−^ (closed circles; n = 9) mice. Left: CEJ-ABC distance measured as in (C). Right: Graph shows total CEJ-ABC distance. (E) CEJ to ABC distances in maxilla of 24-week-old mice fed control (open squares; n = 5) or soft (closed triangles; n = 5) diet since weaning. Left: CEJ-ABC distance measured as in (C). Right: Graph shows total CEJ-ABC distance. (F) CEJ to ABC distances in maxilla of 24-week-old mice fed control (open circles) or hard diet since weaning. Mice fed hard chow pellets received isotype antibody (closed circles, black bars) or anti-IL-17 (red triangles, red bars) i.p. every 5 days. Left: CEJ-ABC distance measured as in (C). Right: Graph shows total CEJ-ABC distance. Data are from two experiments with two to three mice/group. (G) Bar graph shows the CEJ to ABC distances in maxilla of young (white bars) and 24-week-old (black bars) SPF and GF mice. n = 7–13 mice/group. ^∗^p < 0.05, ^∗∗^p < 0.01, as determined by unpaired Student’s t test. ^∗∗∗^p < 0.05 as determined by one-way ANOVA. Error bars represent mean ± SEM. See also Figure S7.

Our data suggested that gingiva mechanical damage was the major driver promoting the accumulation of Th17 cells. As the gingiva is an environment experiencing constant physiological mechanical damage from mastication, we hypothesized that physiologic damage could be a key local cue promoting induction of barrier protective responses. To test this, we induced barrier damage by gingival abrasion and examined IL-17-induced barrier defense mechanisms 5–10 days after abrasion. Gingival damage was sufficient to drive elevated expression of epithelial defensins and neutrophil chemo-attractants (Figure 7A) and led to increased neutrophils in the gingiva (Figure 7B) and local lymph node (Figure S7A). Induction of these responses was sustained after *il6* transcripts in CD45^−^ cells had returned to control levels (Figure S7B). Moreover, induction of this barrier protective program was IL-17 dependent; it was not seen after gingival damage of *il17a*^−/−^ mice (Figure S7C). These data collectively suggested that damage-induced Th17 cell responses promote immune surveillance of the gingival tissue environment.

While mechanical damage-induced Th17 cells could mediate a degree of barrier protection, as Th17 cells are associated with periodontal bone loss we speculated that long-term exposure to these immune mediators could be detrimental and mediate pathogenic consequences at the gingiva. We measured periodontal bone heights (cement-enamel junction [CEJ] to alveolar bone crest [ABC] distances) and documented periodontal bone loss in 24- compared to 8-week-old mice (Figure 7C), suggesting a negative consequence of the damage-induced remodeling of the gingiva cytokine network with age. Moreover, this negative consequence was mediated by IL-17, as reduced bone loss was seen in 24-week-old *il17a*^−/−^ mice (Figure 7D).

We found that physiological mechanical damage was a key driver of this bone loss. We decreased the levels of damage at the gingiva by feeding mice nutritionally matched soft diet from weaning until 24 weeks of age. Reduction in mastication-induced damage resulted in significantly less alveolar bone loss compared to mice fed normal chow (Figure 7E), outlining a key role for damage-induced Th17 cells in this bone loss. Undertaking complimentary experiments, we also placed weanling mice on a hardened irradiated diet, where pellets are harder than normal chow, resulting in increased damage from mastication. Contrasting the mice placed on softer diets, mice on hard diet had elevated frequencies of gingival Th17 cells (Figure S7D). Moreover, these animals exhibited increased bone loss by 24 weeks of age, which was prevented by administration of anti-IL-17 (Figure 7F).

Increased bone loss with age was seen even in GF mice (Figure 7G). 24-week-old GF and SPF mice had similar levels of bone loss, yet when animals were aged to 18 months, bone loss was decreased in GF compared to SPF mice (Figure S7E), underscoring the role of microbe-dependent and -independent factors in driving periodontal bone loss with age.

By promoting Th17 cell effector responses, physiological damage to the gingiva emerges as a key local cue tailoring barrier immuno-surveillance and defense. However, as Th17 cells also promote periodontitis and alveolar bone destruction, gingival mechanical damage also has pathological consequences, promoting elevated bone loss.

## Discussion

Collectively, our data delineate tissue-specific cues responsible for supporting gingival Th17 cells, revealing that unique mechanisms govern CD4^+^ T cell education at this barrier compared to others. Th17 cells are enriched at barriers where they mediate key protective roles (Ivanov et al., 2009, Naik et al., 2012). However, here we have shown that in health few Th17 cells patrol the gingiva in both young adult mice and humans. Nevertheless, this Th17 cell population expanded in the gingiva with age. Although increased Th17 cell differentiation has been reported for T cells from elderly humans and mice (Ouyang et al., 2011), elevated frequencies of gingival Th17 cells occurred by 24 weeks of age, an earlier time point than previously examined and, importantly, one at which increased Th17 cells were not seen at any other site examined, highlighting a specific expansion of this CD4^+^ T cell fate in the gingiva.

The altered gingival CD4^+^ T cell network that emerged by 24 weeks of age led us to first hypothesize that altered commensal bacteria could be driving increased numbers of Th17 cells in older mice. In the GI tract and skin, development of Th17 cells is dependent upon commensal colonization and, as such, in GF animals Th17 cells are dramatically reduced at these sites (Ivanov et al., 2009, Naik et al., 2012). Furthermore, Th17 cells in the GI tract have been shown to be commensal specific (Yang et al., 2014) and therefore bacteria are vital for ensuring that Th17 cells seed these other sites. We have been able to undertake a detailed examination of the oral microbiome of mice and found no significant differences in bacterial biomass, diversity, or composition between young mice lacking gingival Th17 cells and older mice with gingival Th17 cells. Bacterial metabolites have been shown to modulate CD4^+^ T cell differentiation (Atarashi et al., 2008, Smith et al., 2013), so it remained possible that commensal-mediated effects would not be visible at a species level. Therefore, to fully understand the contribution of commensal bacteria to gingival Th17 cells, we examined GF mice. We demonstrated that gingiva Th17 cells were present in GF animals, outlining that a commensal colonization-independent mechanism must ensure their accumulation. These data differ from that at other barriers and suggested that unique mechanisms operate in the gingiva to control CD4^+^ T cell effector function. Indeed, alongside Th17 cells, commensal colonization did not affect gingival Treg cell frequencies, which is again different from other barriers. Our data contrasted what has been reported for Th17 cell development in the tongue, a physiologically distinct barrier in the oral cavity (Conti et al., 2014). Tongue-resident Th17 cells develop in a commensal-dependent manner, further highlighting the novel pathways of gingival CD4^+^ T cell education.

Having demonstrated that gingiva Th17 cells develop independently of commensal colonization, we sought to understand the cues driving differentiation of these disease-relevant cells. Induction of the Th17 cell fate is driven by the cytokines transforming growth factor beta (TGF-β), IL-1β, IL-23, and IL-6 (Bettelli et al., 2006, Harrington et al., 2005, Mangan et al., 2006, Veldhoen et al., 2006). Again contrasting other barrier sites, we showed that gingival Th17 cells develop in the absence of IL-1 signaling. Key roles for IL-1 in supporting the development and maintenance of Th17 cells have been shown in both the skin and GI tract (Coccia et al., 2012, Naik et al., 2012, Shaw et al., 2012). Yet it is likely the IL-1 at these sites is produced in response to the microbiota (Naik et al., 2012, Seo et al., 2015). Subsequently, we determined that IL-6 signals were vital for gingival Th17 cell development. IL-6 is not required for Th17 cell development in the skin (Naik et al., 2012) and this has also been suggested for the GI tract (Shaw et al., 2012). However, a role for IL-6 in GI Th17 cell development has been reported (Persson et al., 2013). Yet, this study demonstrated that CD11b^+^CD103^+^ dendritic cells were the key source of IL-6, whereas our data showed that epithelial cells were an important gingival source of this cytokine, again indicating a differential control of Th17 cell development. Our data from mixed bone marrow chimeras, utilizing wild-type and *il6ra*^−/−^ bone marrow, further highlighted that IL-6 signals were vital for IL-17 production by gingival CD4^+^ T cells.

To elucidate the stimulus capable of driving gingival Th17 cell accumulation in the absence of endogenous microbiota, we queried which signals present in the oral environment could support acquisition of the Th17 cell fate. A unique, tissue-specific signal in the oral environment is on-going mastication. We demonstrated that this physiological damage participated in the expansion of gingival Th17 cells in an antigen- and IL-6-dependent manner. We suggest that mechanical damage due to mastication, over the period of 24 weeks, led to sufficient levels of IL-6 to promote elevated frequencies of gingival Th17 cells. Mechano-sensing by cells triggers multiple cellular responses; indeed, mechanical stretch promotes a pro-inflammatory response from a plethora of cells including osteoblasts, fibroblasts, and endothelial and epithelial cells. This pro-inflammatory response includes IL-6 production (Fukuno et al., 2011, Kobayashi et al., 2003, Skutek et al., 2001). Our data show activity of a similar pathway in the gingiva where epithelial cells produce IL-6 in response to mechanical damage. As this IL-6 affects T cell fate, we consequently have outlined a novel tissue-specific cue shaping CD4^+^ T cell function at a barrier site.

Here we have also demonstrated that local damage not only promotes Th17 cells but also contributes to the potentiation and exacerbation of local oral immunity. In young mice, presence of damage-induced gingival Th17 cells led to elevated barrier protective responses including increased anti-microbial peptides and neutrophil chemo-attractants, suggesting that damage is a physiologic cue shaping homeostatic immunity at this barrier. However, although physiologic damage enhanced barrier protective mechanisms, we also show that with age, these elevated responses can have a detrimental effect. Both elevated Th17 cells and neutrophils (Eskan et al., 2012, Moutsopoulos et al., 2014) are causative in periodontitis, as are dysbiotic microbial communities that stimulate aberrant inflammatory responses promoting the disease in mouse models (Hajishengallis et al., 2011) and in susceptible patients (Darveau, 2010, Lamont and Hajishengallis, 2015). Our data show that gingival mechanical damage acts as an amplifier of this oral inflammatory response and contributes to pathogenic bone loss. Consistent with this concept, we demonstrated that age-induced bone loss can be altered by modulating levels of mastication. This concept of mastication-induced damage contributing to Th17-cell-driven bone loss with age can conceivably be translated in the human setting. In humans, physiologic damage from mastication is an on-going stimulus that could potentially be a contributing factor to the age-related Th17 cell increase we observe and progression to periodontitis, a disease occurring at higher incidence with age. In fact, it has been clinically observed, and shown in animal models, that elevated mechanical damage from biting, known as occlusal forces (Hallmon, 1999), leads to increased periodontal bone loss in settings of periodontitis, a phenomenon termed “secondary trauma from occlusion” (Lindhe and Svanberg, 1974, Polson et al., 1976). Our data provide a biological basis for these clinical observations, identifying mechanical damage as a tissue-specific cue supporting Th17 cell responses, which can exaggerate periodontal bone loss.

In sum, although a full understanding of host-commensal cross-talk in calibrating steady-state immunity in the gingiva remains to be determined, here we demonstrate a commensal colonization-independent mechanism supporting Th17 cells in the gingiva; this starkly contrasts developmental requirements for these cells at other barrier sites. Instead, gingival mechanical damage promoted the proliferation of Th17 cells, highlighting physiological damage as a key local cue tailoring immuno-surveillance at this barrier. These data provide insight into tissue training of immunity at the gingiva, outlining that different signals dominate in tailoring immune responses at distinct barrier sites and suggesting unique ways to modulate pathogenic gingival Th17 cell responses.

## Experimental Procedures

### Mice

C57BL/6, *il1r1*^−/−^, *OT-IIxrag*^−/−^, *il17a*^−/−^, and *il12b*^−/−^ mice were from Taconic or the NIAID-Taconic exchange. C57BL/6 mice were also purchased from the Jackson Laboratory or Harlan. Other gene-deficient animals were *il23a*^−/−^ (from G. Trinchieri at NCI, NIH), *il6*^−/−^ (from S. Anderton at University of Edinburgh), *il1a/b*^−/−^(Horai et al., 1998), and *il6ra*^−/−^ (from S. Jones at University of Cardiff). Germ-free mice were from the University of Manchester Gnotobiotic Facility, the NIAID Microbiome Project Gnotobiotic Animal Facility, the NCI Gnotobiotic Animal Facility at Frederick, or the University of McMaster Germ Free Facility. All experiments were approved by appropriate governing bodies and performed according to local rules.

### Human Samples

All subjects signed informed consent and enrolled on an IRB-approved protocol (ClinicalTrials.gov #NCT01568697) at the NIH Clinical Center. For details on patient inclusion, see Supplemental Experimental Procedures and Figure S1B.

### Generation of Mixed Bone Marrow Chimeras

Bone marrow from *il6ra*^*−/−*^ and WT mice was T cell depleted using microbeads (Miltenyi Biotec). CD45.1^+^CD45.2^+^ animals were lethally irradiated and reconstituted with equal numbers of WT (CD45.1) and *il6ra*^*−/−*^ (CD45.2) bone marrow cells. After transfer, mice were aged to 24 weeks.

### Preparation of Single-Cell Suspensions

For mice, gingiva was dissected and digested for 50 min at 37°C with CollagenaseIV (GIBCO) and DNase (Sigma) as previously described (Dutzan et al., 2016a). Single-cell suspensions were obtained from small intestinal lamina propria after digestion at 37°C with Liberase TL (Roche) and from the skin after digestion as previously described (Naik et al., 2012). For details on tape-striping experiments, see Supplemental Experimental Procedures. For humans, gingival biopsies were processed as previously described (Dutzan et al., 2016b).

### Flow Cytometry

Single-cell preparations were stained with antibodies from eBioscience, BD Biosciences, and Biolegend. Cytokines, Foxp3, Ki67, and Bcl-2 were stained using the eBioscience Fix/Perm kit. Cytokines were also stained in buffer containing 0.5% Saponin (Sigma). Dead cells were excluded by use of a Live/Dead fixable dye (Biolegend). Samples were acquired using a Fortessa (BD Biosciences) and analyzed with FlowJo software (Treestar). Cell sorting was performed using an Influx (BD Biosciences).

### Ex Vivo Re-stimulation for Cytokine Detection

Cells were stimulated with 50 ng/mL PMA (Sigma-Aldrich) and 5 μg/mL ionomycin (Sigma-Aldrich) in the presence of GolgiPlug (Brefeldin A; BD Biosciences). After 3.5–4 hr, cells were stained for flow cytometric analysis.

### Real-Time RT-PCR

RNA was obtained from cells with an RNeasy Mini or Micro kit (QIAGEN) or from gingival tissues using Trizol and cDNA synthesized with Superscript reverse transcription kit (Invitrogen/Life Technologies). Quantitative real-time PCR was done with TaqMan primers/probes (Applied Biosystems) or with SYBR green qPCR super mix (Invitrogen/Life Technologies). Results were normalized to *hprt* expression.

### Oral Microbiome Evaluation via 16S rRNA Gene Sequencing and qPCR

For microbiome analyses, the murine oral cavity was sampled for 30 s using sterile ultra-fine swabs. For detailed descriptions of DNA isolation, sequencing, and analysis, see Supplemental Experimental Procedures.

### Gene Expression Analysis by NanoString

The nCounter analysis system (NanoString Technologies) was used to screen gene expression. For further details, see Supplemental Experimental Procedures.

### Scratch Assay

Human oral keratinocytes (HOKs) (ScienCell Research Laboratories) are isolated from human oral mucosa and propagated in Oral Keratinocyte Growth Supplement (ScienCell Research Laboratories) until 90% confluent and a scratch assay performed with a 200 μL pipette with or without indicated inhibitor.

### Bone Loss Measurements

Periodontal bone heights were assessed after defleshing and staining with methylene blue. The distance between the cemento-enamel junction and alveolar bone crest (CEJ-ABC distance) was measured at six predetermined sites as previously described (Eskan et al., 2012) and combined to give a total CEJ-ABC distance.

### Statistics

p values were determined with Student’s unpaired t test unless otherwise stated.

## Author Contributions

J.E.K. and N.M.M. conceived of, designed, and supervised the research, analyzed data, and wrote the paper. J.E.K., N.D., L.A., H.B., T.G.-W., T.Z.-M., M.E.F., N.B., H.L., K.W., G.C., and T.J.B. performed the experiments. L.A., B.-Y.H., and P.I.D. undertook 16S sequencing and analysis. L.B. and N.M.M. secured human samples. D.M.E.B., M.S.L., S.A.J., G.T., P.I.D., and Y.B. provided tools and/or reagents and key scientific input.

## Supplemental Information

Document S1. Figures S1–S7 and Supplemental Experimental Procedures

Document S2. Article plus Supplemental Information
