# Supplementary material for: On-going Mechanical Damage from Mastication Drives Homeostatic Th17 Cell Responses at the Oral Barrier
Source: Immunity. 2017 Jan 17;46(1):133–47. doi: 10.1016/j.immuni.2016.12.010 (PMC5263257; doi:10.1016/j.immuni.2016.12.010)
Supplement: Document S1. Figures S1–S7 and Supplemental Experimental Procedures [file mmc1.pdf]

Immunity, Volume *46*

Supplemental Information

On-going Mechanical Damage from Mastication Drives Homeostatic Th17 Cell Responses at the Oral Barrier

Nicolas Dutzan, Loreto Abusleme, Hayley Bridgeman, Teresa Greenwell-Wild, Tamsin Zangerle Murray, Mark E. Fife, Nicolas Bouladoux, Holly Linley, Laurie Brenchley, Kelly Wemyss, Gloria Calderon, Bo-Young Hong, Timothy J. Break, Dawn M.E. Bowdish, Michail S. Lionakis, Simon A. Jones, Giorgio Trinchieri, Patricia I. Diaz, Yasmine Belkaid, Joanne E. Konkel, and Niki M. Moutsopoulos

A

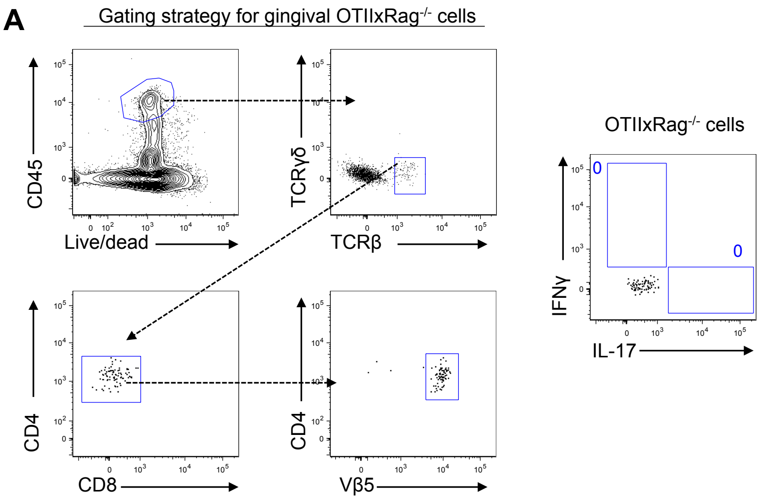

B

Inclusion criteria for Healthy Volunteers

Adults 18-60 years of age who are systemically healthy

General Exclusions for Healthy Volunteers

1. Smoking/Tobacco use
2. HIV, Hepatitis B or C positive
3. History of diabetes and/or HbA1C level >6%
4. More than 3 hospitalizations in the last 3 years
5. History of systemic illness or malignancy except for localized basal or squamous cell carcinoma of the skin
6. Pregnant or lactating
7. In the 3 months before study enrollment, have used any of the following:
   - Systemic (intravenous, intramuscular) or oral antibiotics
   - Oral, intravenous, intramuscular, intranasal, or inhaled corticosteroids or other immunosuppressant
   - Cytokine therapy
   - Methotrexate or immunosuppressive chemotherapeutic agents
   - Large doses of commercial probiotics

Oral health Exclusions for Healthy Volunteers:

1. Signs/symptoms of xerostomia
2. Active Infection
3. Missing teeth (excluding 3d molars)
4. Diagnosis of Periodontal Disease
5. Presence or history of mucosal lesions
6. Active caries or significant caries history

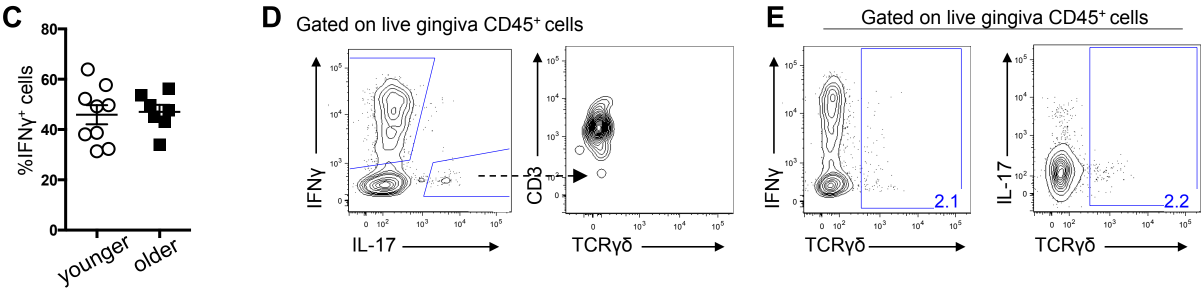

Supplementary Figure 1.

Supplementary Figure 1. Related to Figure 1.
(A) Cytokine production by gingiva $CD4^+$ T cells was examined in 24-week old OTIIxRag$^{-/-}$ mice. Representative FACS plots show gating strategy for examining transgenic T cells and IFNγ and IL-17 staining in these cells. Data representative of 6 mice from 3 separate experiments.
(**B**) Inclusion and Exclusion Criteria for Healthy Volunteers.
(**C-E**) Single cell preparations of human gingiva were stimulated with PMA and ionomycin. (**C**) Bar graph showing frequency of gingival IFNγ$^+$ cells in healthy individuals who were 18-25 or 40-50 years of age. (**D**) Representative FACS plots show (Left) IFNγ versus IL-17 staining gated on Live, $CD45^+$ cells in gingiva from healthy individuals and (Right) expression of CD3 and TCRγδ gated on live, $CD45^+$IL-17$^+$ cells. (**E**) Representative FACS plots show (Left) IFNγ versus TCRγδ gated on live, $CD45^+$ cells and (Right) IL-17 versus TCRγδ gated on live, $CD45^+$ cells.
Results are expressed as mean±SEM**.**

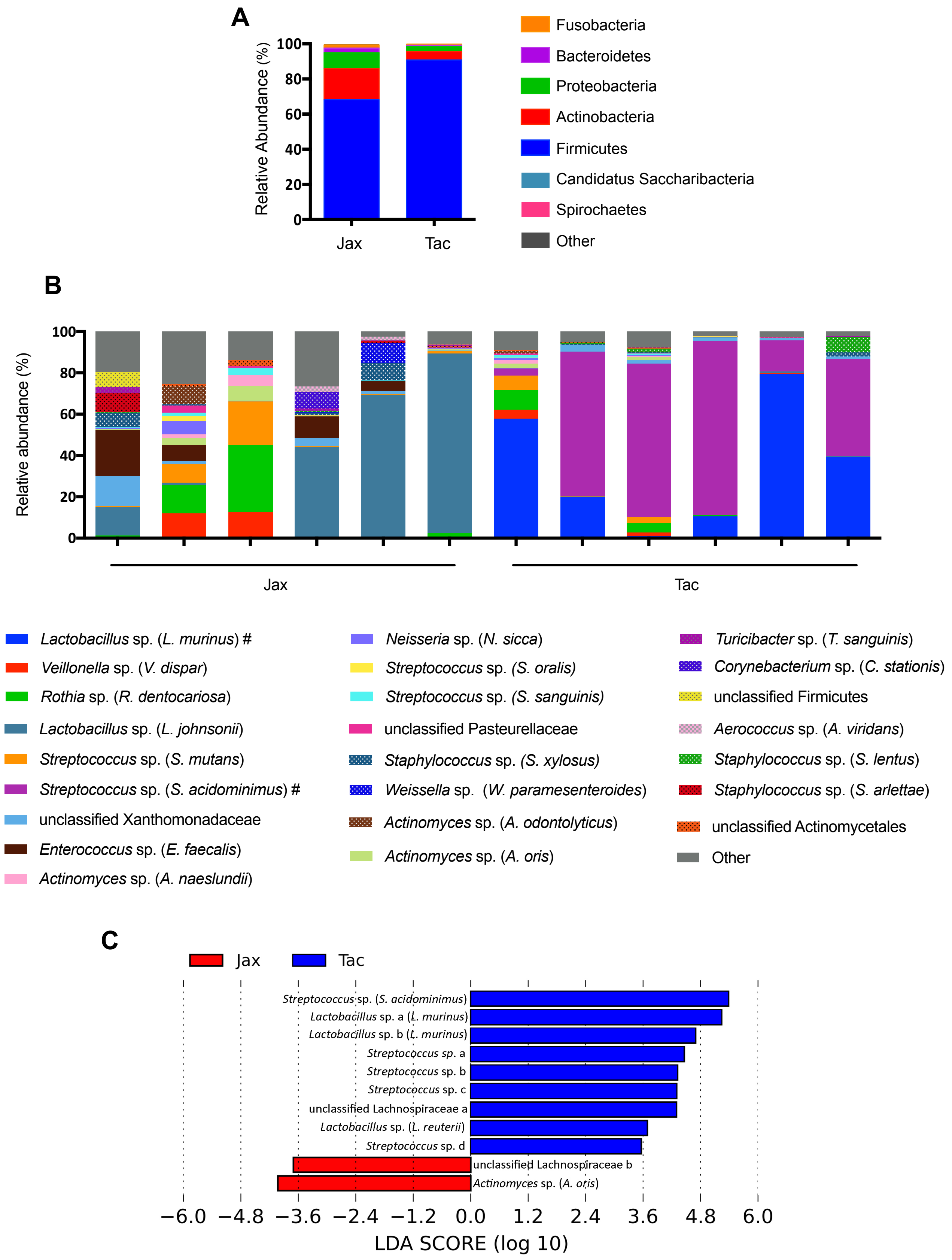

Supplementary Figure 2.

Supplementary Figure 2. Related to Figure 2.

(**A**) Bar graph depicts mean relative abundance of the main phyla found in the oral microbiome of Jax and Tac mice. No differences were observed as determined by LEfSe analysis.

(**B**) Bar graph shows the most abundant OTUs (2% of the reads in at least one sample) in the oral microbiota of Jax and Tac mice. # indicates that the relative abundance of a given OTU was significantly higher in Tac mice, according to LEfSe analysis.

(**C**) Bar graph depicts all discriminant OTUs as determined by LEfSe.

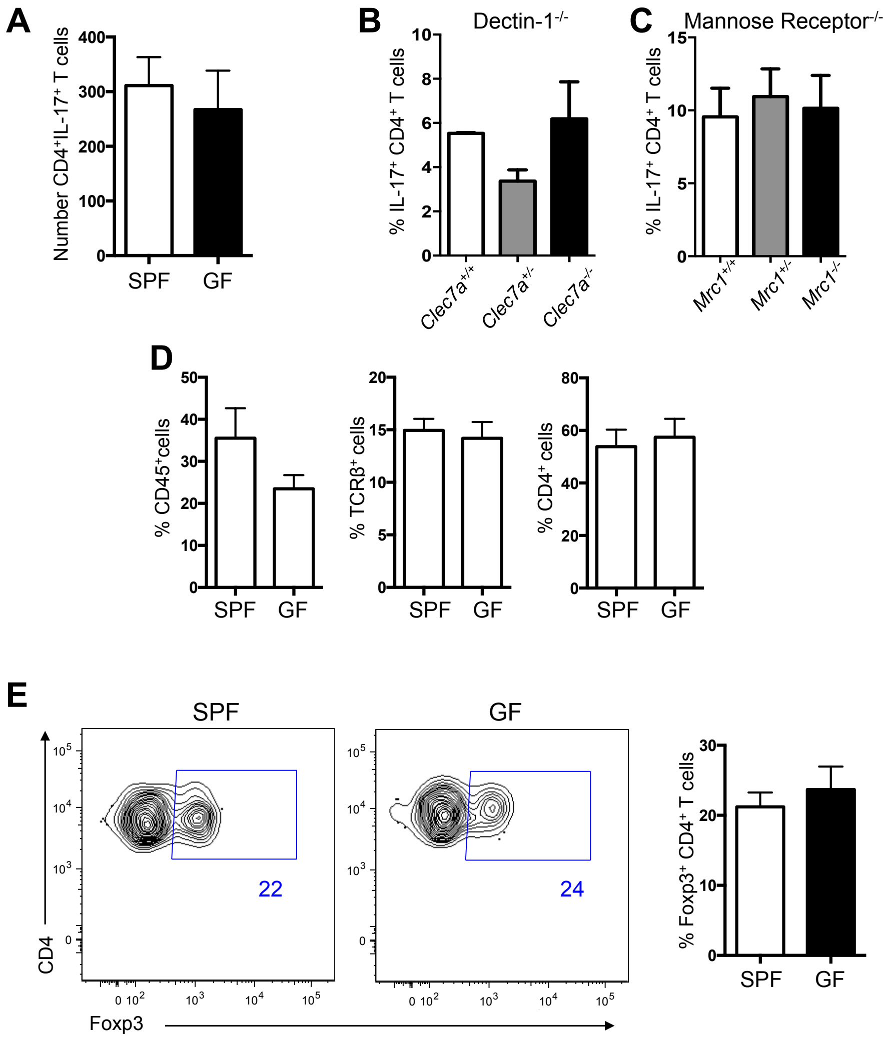

Supplementary Figure 3. Related to Figure 3.

(A) Bar graph shows total number of CD4$^+$IL-17$^+$ T cells in gingiva of SPF and GF mice (n=5).

(**B, C**) Bar graphs show percent of gingiva IL-17$^+$CD4$^+$ T cells in (**D**) Dectin-deficient animals (*Clec7a*$^{-/-}$, n=3) and controls (n=2 for $^{+/+}$ and n=3 for $^{+/-}$) and (**E**) Mannose receptor-deficient animals (*Mrc1*$^{-/-}$, n=5) and controls (n=5 for $^{+/+}$ and n=6 for $^{+/-}$).

(D) Bar graphs showing percentages of gingival CD45$^+$ cells within the live gate, TCRβ$^+$ cells within the CD45$^+$ gate and CD4$^+$ T cells within the TCRβ$^+$ gate in age-matched SPF (n=6) and GF (n=7) mice.

(**E**) Representative FACS plots and bar graph showing the frequencies of Foxp3$^+$ Tregs at the oral barrier of SPF (n=6) and GF (n=5) mice.

Data from 2-3 experiments. Results are expressed as mean±SEM**.**

Supplementary Figure 3.

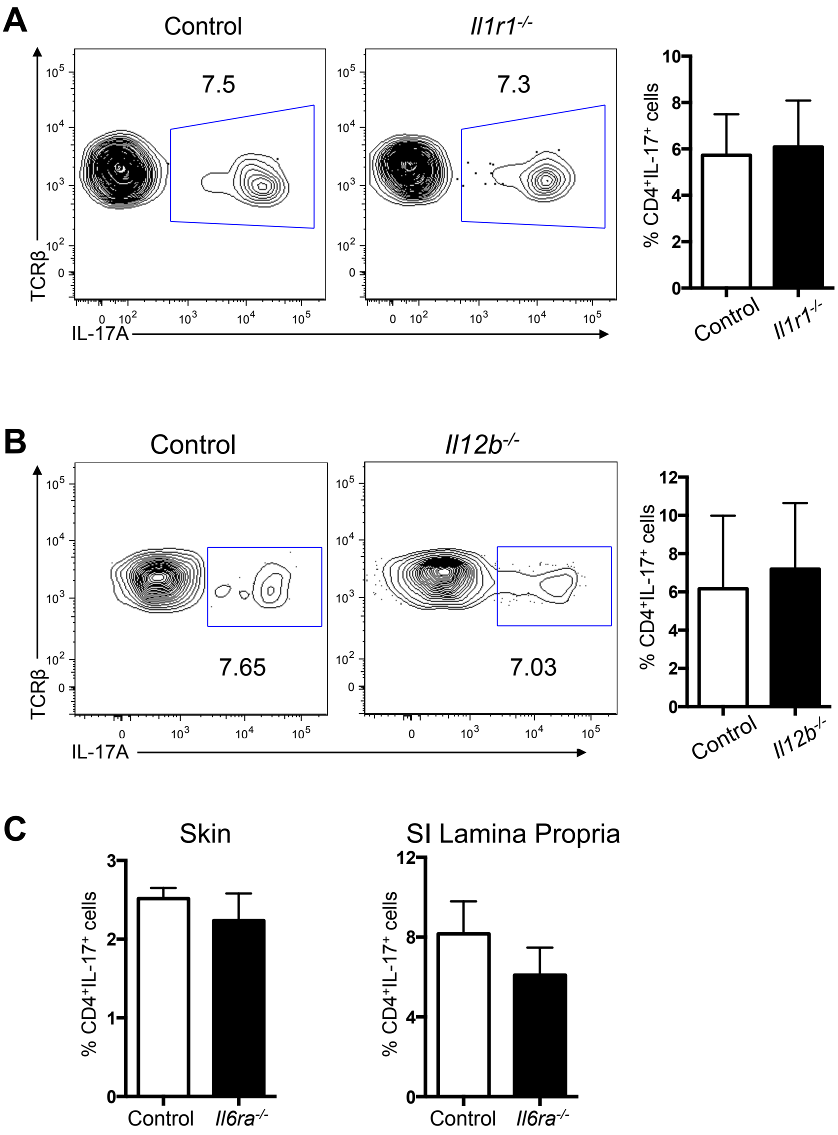

Supplementary Figure 4. Related to Figure 4.

(**A,B**) Representative FACS plots and bar graphs showing IL-17$^+$ TCRβ$^+$ CD4$^+$ cells in gingiva of 24-week old **(A)** control (control; n=5) and *Il1r1$^{-/-}$* (KO; n=4) mice, and **(B)** control (control; n=4) and *Il12b$^{-/-}$* (KO; n=3) mice. Data from 2-3 experiments.

(C) Chimeric mice comprised of wild-type CD45.1$^+$ and *Il6ra$^{-/-}$* CD45.2$^+$ bone marrow were generated and skin and small intestinal lamina propria CD4$^+$ T cell cytokine production examined at 24-weeks of age. Bar graph shows frequency of (left) skin and (right) gut IL-17$^+$CD4$^+$ T cells in wild-type control and *Il6ra$^{-/-}$* bone marrow compartments. Data representative of 3 independent experiments with 2-4 mice per group.

Results are expressed as mean±SEM**.**

Supplementary Figure 4.

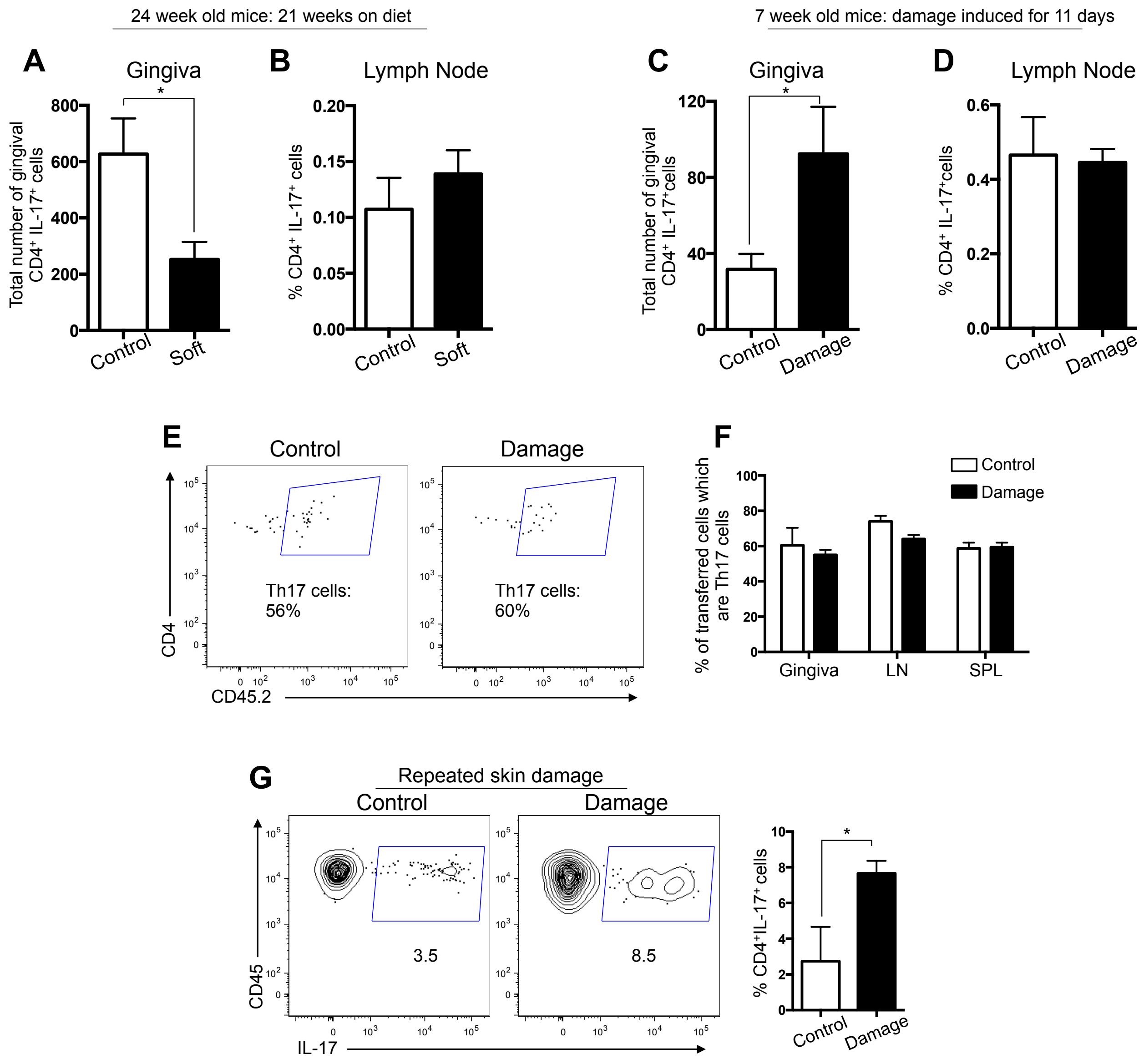

Supplementary Figure 5.

Supplementary Figure 5. Related to Figure 5.

(**A,B**) Mice were fed control diet (white bars) or a soft diet (black bars) from weaning and Th17 cells examined when mice reached 24-weeks of age. Bar graphs show (**A**) total number of IL-17$^+$CD4$^+$ T cells in the gingiva and (**B**) the frequency of IL-17$^+$CD4$^+$ T cells in the oral barrier draining lymph nodes from 2-3 separate experiments combined.

(**C,D**) Young mice underwent gingival barrier damage every other day for 11 days after which Th17 cells were examined. Bar graphs show (**C**) total number of IL-17$^+$CD4$^+$ T cells in the gingiva and (**D**) the frequency of IL-17$^+$CD4$^+$ T cells in the oral barrier draining lymph nodes. Data from 2-3 experiments.

(E, F) Young CD45.2$^+$ C57BL/6 mice received an i.v. transfer of *in vitro* differentiated CD45.1$^+$CD45.2$^+$ Th17 cells along-with CD45.1$^+$ non-polarized, activated CD4$^+$ T cells (Th0 cells). Following transfer mice were left untreated or underwent gingival barrier damage every other day for 11 days after which Th17 cells were examined. (**E**) FACS plot show the percentage of CD4$^+$CD45.1$^+$ transferred cells which are CD45.2$^+$ (i.e. Th17 polarized), in the gingiva of control (left) and damaged (right) mice. (**F**) Bar graph shows percent of transferred cells in the gingiva which are Th17 polarized. Data are representative of 2 experiments with 3-4 mice per group.

(G) The back skin of wild-type mice was tape-stripped every other day for 8 days prior to examination of T cell cytokine production. Representative FACS plots and bar graph show frequency of skin CD4$^+$ T cells which are positive for IL-17 staining in control mice and mice which experienced repeated tape-stripping. Data are representative of 2 experiments with 2-3 mice per group.

*$p<0.05$ as determined by unpaired students *t*-test. Results are expressed as mean±SEM**.**

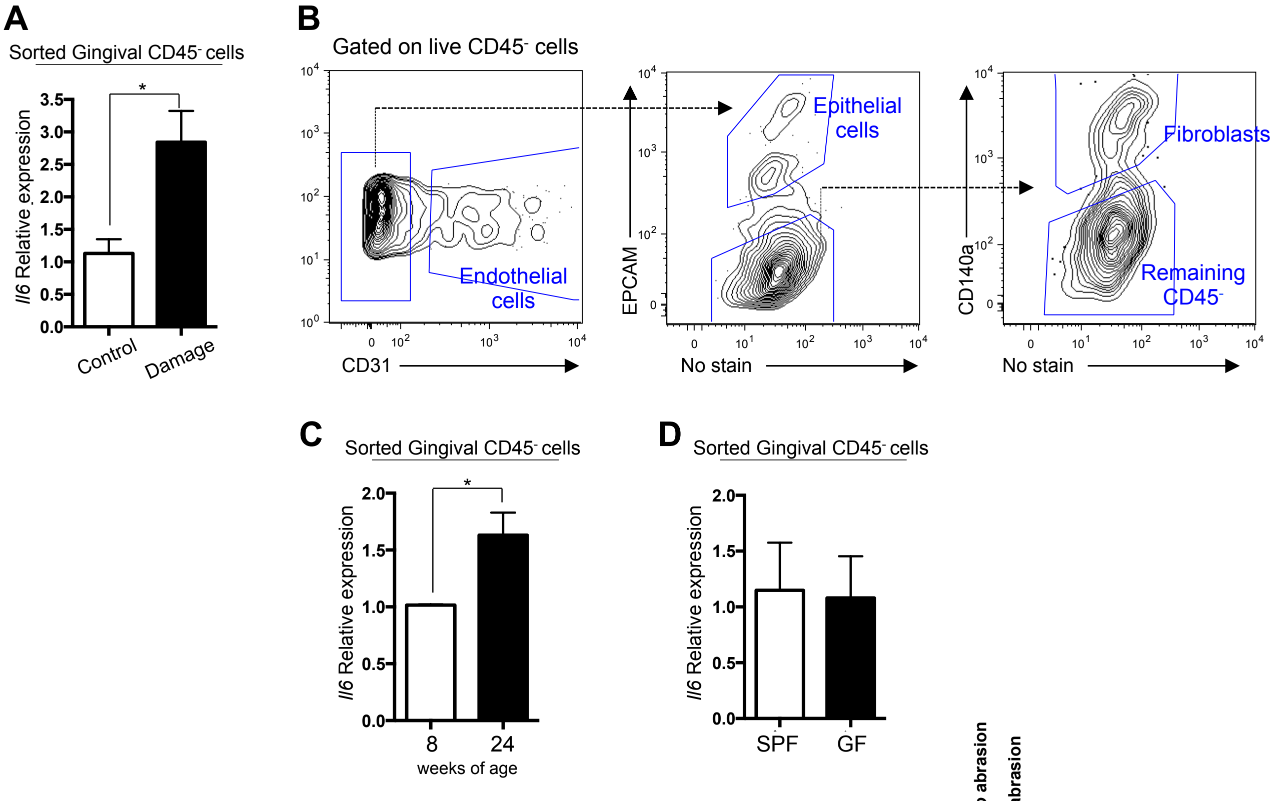

E

Gene ID	Fold Change
EGR1	306.13
PTGS2	28.699
EGR2	24.05
IL20	21.87
SOCS3	17.551
KLRC3	17.49
CXCL2	17.209
CSF2	16.4
SLAMF7	16.4
IL6	15.661
BATF3	15.31
JAK3	15.31
PDGFB	15.31
TCF7	15.31
CYBB	13.12

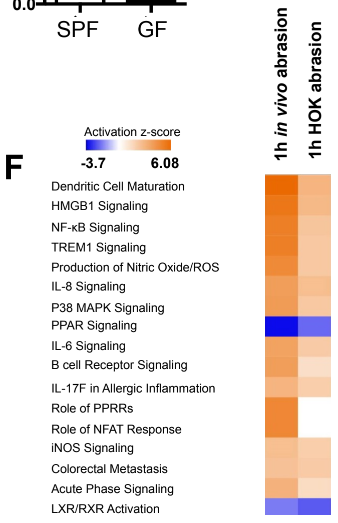

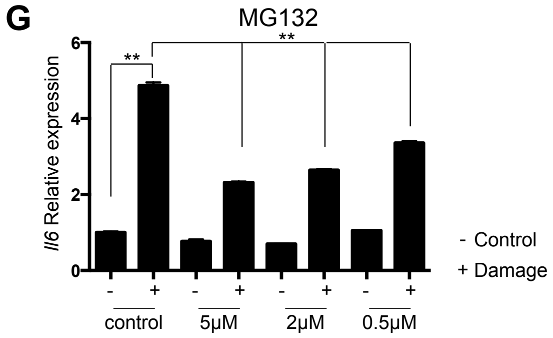

Supplementary Figure 6.

Supplementary Figure 6. Related to Figure 6.

(**A**) $CD45^-$ cells were sorted from the gingiva of young control mice and those which experienced repeated gingival damage. Bar graphs show *il6* expression determined by qPCR. Expression in $CD45^-$ cells sorted from mice that experienced gingiva damage (black bar) is shown relative to that in controls (white bar). Data is from 3 separate sets of sorted cells.

(**B**) Gating strategy to identify subsets of $CD45^-$ cells in the gingiva. Initial FACS plot is gated on $CD45^-$Live cells.

(C,D) $CD45^-$ cells were sorted from the gingiva (**C**) 8- and 24- week old mice and (**D**) age-matched GF and SPF mice. Bar graphs show *il6* expression determined by qPCR. Expression in $CD45^-$ cells in all graphs is shown as the black bar relative to the white bar. For each graph, data is from 3 separate sets of sorted cells.

(**E**) Nanostring immune gene array. List of 15 top genes upregulated in Human Oral Keratinocytes 1 hour after *in vitro* damage (scratch assay).

(F) Common pathways activated 1h-post damage *in vivo* in mice (mouse gingival abrasion) and *in vitro* in human cells (Human Oral Keratinocytes scratch assay).

(**G**) *In vitro* scratch assays on Human Oral Keratinocytes were undertaken in the presence or absence of the NFkB inhibitor MG132 or DMSO control. RNA expression was examined after 4 hours; graph shows *il6* levels in damaged cells relative to that in un-damaged control. Data representative of 3 independent experiments.

*$p \leq 0.05$ as determined by unpaired students *t*-test. **$p \leq 0.05$ as determined by ANOVA. Results are expressed as mean±SEM**.**

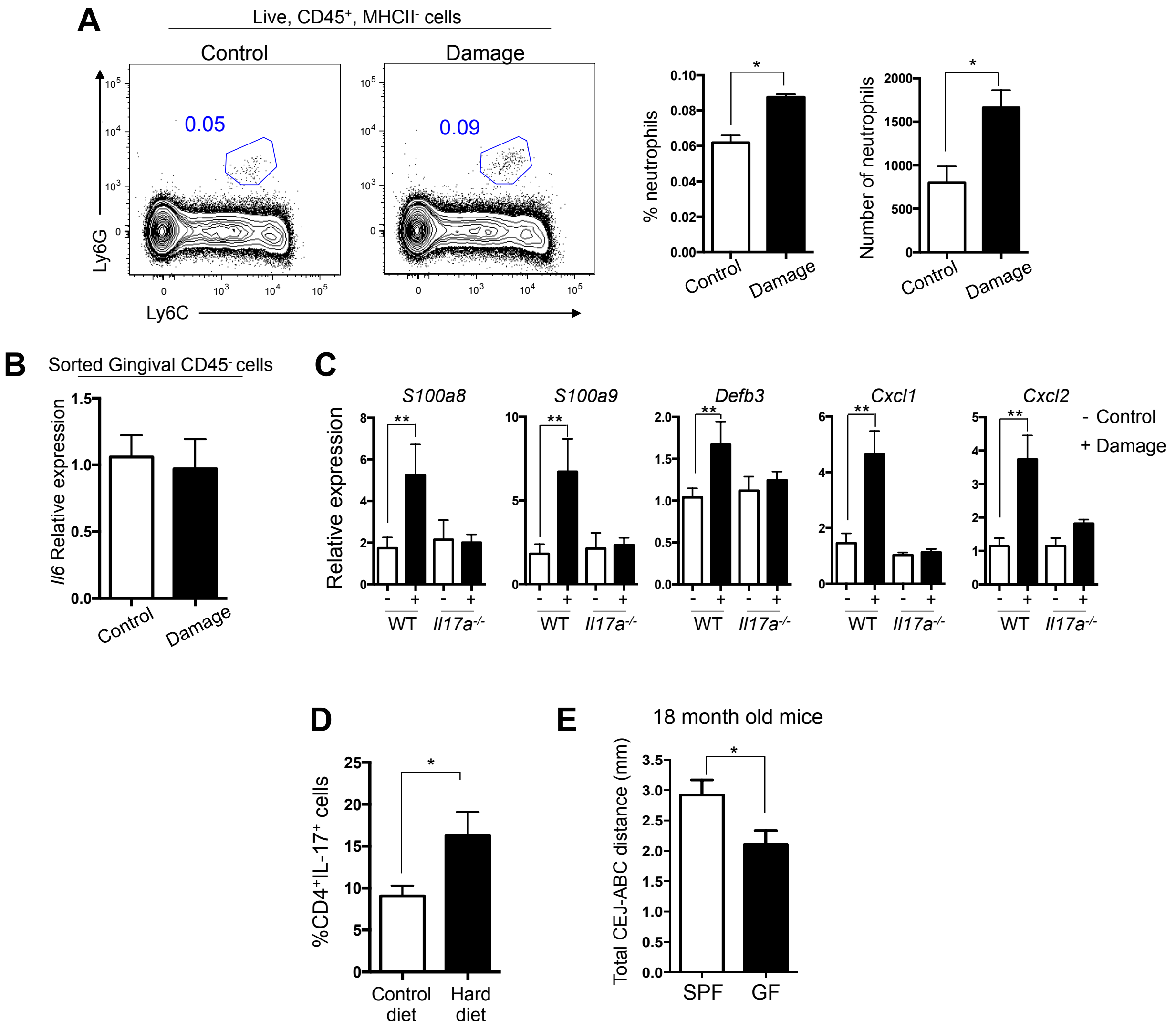

Supplementary Figure 7.

Supplementary Figure 7. Related to Figure 7.

(**A,B**) 8-week old mice were either untreated (control) or experienced gingival damage every other day for 11 days. Following this mice were left untreated for 5-10 days before gingiva and draining lymph nodes were examined. **(A)** Neutrophils were examined in the oral barrier draining lymph node, representative FACS plots show Ly6C versus Ly6G staining on live $CD45^{+}MHCII^{-}$ cells. Bar graphs show percent and number of neutrophils. (**B**) $CD45^{-}$ cells were FACS sorted from the gingiva of these control or damage mice and *il6* expression determined by qPCR. Graph shows expression in $CD45^{-}$ cells of mice experiencing damage relative to that in control gingiva. Data representative of 2-3 separate experiments.

(C) Wild-type control (WT) and *Il17a*$^{-/-}$ mice were either untreated (-) or experienced gingival damage (+) every other day for 11 days. Following this mice were left untreated for 5-10 days before gingiva harvested and expression levels of the indicated genes examined. Graphs show expression levels in mice which experienced gingival damage (+) compared to untreated mice (-) of the same genotype. n=4-6 mice per group and **$p<0.05$ as determined by paired students *t*-test.

(**D**) Mice were fed control diet (white bars) or hard chow pellets (black bars) from weaning and the oral barrier examined at 24 weeks of age. Bar graphs show frequencies of IL-$17^{+}CD4^{+}$ T cells in the gingiva. Data are from 2 experiments with 2-3 mice per group.

(E) Bar graph shows the total Cemento-Enamel Junction (CEJ) to Alveolar Bone Crest (ABC) distances in maxilla of 18-month old SPF (white bars) and GF (black bars) mice. (n=4-6 mice per group).

*$p<0.05$ as determined by unpaired students *t*-test. Results are expressed as mean±SEM**.**

Supplemental Experimental Procedures

Human Samples

For inclusion in the healthy volunteer group, subjects reported good general health and had no significant medical history, see **Fig. S1**. All subjects were evaluated for the presence of active infections, mucosal lesions and presence/history of periodontal disease. Subjects were examined with full mouth evaluation of bone loss and inflammation; probing depths (PD), attachment loss (CAL) and bleeding on probing (BOP). For inclusion subjects had no sites with significant bone loss (PD/CAL>3mm), BOP<10% and absence of visible gingival inflammation. Gingival Collar biopsies from molar teeth of ~4mm length by 2mm width were taken from each subject and used in this study.

Mouse treatment protocols

Mice were administered the following treatments where indicated; 100µg/mouse anti-IL-17 (clone 17F3; BioXCell), 100µg/ml FTY720 (Sigma) i.p., 1.5% Ovalbulmin (GradeV, Sigma) in the drinking water, or 1mg/mouse Ovalbulmin was topically applied to the gingiva with or without gingival damage.

Tape Stripping of mouse back skin

Mouse back was stripped of hair and then tape-strip epilated every other day for 8 days using epilating strips. Back skin was then dissected and single cell suspension obtained following digestion in dispase II (Roche).

In vitro Th17 cell polarisation

$CD4^+$ T cells were isolated from spleens and lymph nodes of congenic animals using magnetic beads (Miltenyi Biotec). Cells were cultured with plate bound anti-CD3

(5μ/ml) and soluble anti-CD28 (2μg/ml) (Th0) or anti-CD3, anti-CD28, TGFβ1 (2ng/ml) and IL-6 (50ng/ml) (Th17). After 5 days of culture cells were washed and counted prior to i.v. transfer into C57BL/6 hosts.

Gene expression analysis by NanoString

Gingival tissues were dissected from age-matched GF and SPF control animals and total RNA was hybridized with reporter and capture probes for a murine immunology panel (NanoString Technologies) as per manufacturer's instructions. Data were normalized to housekeeping genes and spiked positive controls. Transcript counts less than the mean of the negative control transcripts plus 1 SD for each sample were considered as background. Data were analysed using Ingenuity Pathway Analysis software (IPA, Qiagen).

Oral microbiome evaluation via 16S rRNA gene sequencing and qPCR

DNA was isolated using the DNeasy Blood and Tissue kit (Qiagen) as described previously (Abusleme et al., 2013). Total 16S rRNA copy numbers were determined as previously described(Nadkarni et al., 2002). SFB SYBR Green real-time PCR was conducted using SFB specific primers SFB736 forward 5'-GACGCTGAGGCATGAGAGCAT-3' and SFB844 reverse 5'-GACGGCACGGATTGTTATTCA-3'. For 16S rRNA gene sequencing, amplicon libraries were generated using fusion primers containing universal primers 8F 5'-AGAGTTTGATCMTGGCTCAG-3' and 361R 5'-CYIACTGCTGCCTCCCGTAG-3'(Sundquist et al., 2007), which span the V1-V2 regions. Primers also contained 5' and 3' linker sequences, index identifiers and heterogeneity spacers as previously described(Fadrosh et al., 2014). Amplicons were prepared in duplicate PCR reactions containing 0.3μM of each primer, 200mM of each dNTP, 1.5mM $MgSO_4$, and 0.625U of Platinum® Taq DNA Polymerase High Fidelity (Invitrogen). Amplification conditions included an initial denaturation step at 95°C for 3 min, 35

cycles of 95°C for 30s, 50°C for 30s and 72°C for 60s, followed by a final elongation step at 72°C for 9 min. PCR products and negative DNA extraction and PCR control reactions were purified using Agencourt AMPure XP reagents, quantified, pooled and sequenced using the MiSeq Reagent Kit v3 (2x300 cycle) (Illumina). 16S rRNA reads were processed in Mothur (Schloss et al., 2009). Unassembled raw reads were first quality-trimmed using a sliding window approach (window size 50bp, quality score >35). Reads were then assembled, primers, spacers and indices trimmed and contigs filtered to include only sequences between 200-400bp and no ambiguous base calls. Sequences were further processed following a standard pipeline(Kozich et al., 2013). Sequences were classified using Mothur's version of the Ribosomal Database Project classifier (Wang et al., 2007) with a cutoff=80. For Operational Taxonomic Unit (OTU) analyses, sequences were clustered using a 97% similarity cutoff. OTUs were classified up to genus level based on the consensus taxonomy using the default cutoff (51%). To further inform OTU taxonomy, the representative sequence from each OTU was compared by BLAST to the NCBI 16S rRNA sequence database and the top match (with at least 97% similarity and coverage) is reported in parenthesis as part of the OTU name. Differences in relative abundance of taxa between young and old mice were determined via paired-sample Wilkoxon Rank tests with adjustments for multiple testing done via the Benjamini-Hochberg false discovery rate method. Differences between Tac and JAX mice were evaluated with LEfSe (Segata et al., 2011) using 0.05 as the alpha value for the factorial Kruskal-Wallis test. Beta diversity was measured with the ThetaYC distance for comparison of communities based on global structure. Principal Coordinates Analysis (PCoA) was performed in mothur using OTU-level ThetaYC distances. Graphs were visualized using the rgl application within R (http://www.r-project.org) and AMOVA was used to test for differences in community structure.
